# Impact of Arsenite on Transient and Persistent Histone H3 Modifications and Transcriptional Response

**DOI:** 10.1021/acs.chemrestox.5c00312

**Published:** 2026-01-02

**Authors:** Tatjana Lumpp, Hassan Hijazi, Sandra Stößer, Eda Tekin, Lara Brunner, Franziska Fischer, Sabine Brugière, Delphine Pflieger, Andrea Hartwig

**Affiliations:** † Institute of Applied Biosciences, Department of Food Chemistry and Toxicology, 150232 Karlsruhe Institute of Technology (KIT), Adenauerring 20a, 76131 Karlsruhe, Germany; ‡ 27015 University Grenoble Alpes, INSERM, CEA, UA13 BGE, CNRS, CEA, UAR2048, 38000 Grenoble, France

## Abstract

Arsenite-contaminated groundwater poses a major health concern affecting millions of people. Chronic exposure to elevated levels of inorganic arsenic is implicated in carcinogenesis, with impaired DNA repair and dysregulated DNA and histone modifications as key factors. Using human A549 lung carcinoma cells, we investigated the persistence of acute arsenite-induced cellular stress at the epigenetic and transcriptional levels after 24 h of exposure to 1–25 μM NaAsO_2_, reflecting low to high acute exposure scenarios, followed by a 48 h arsenite-free postincubation period. The primary objective was to analyze alterations in acetylation and methylation marks on both bulk histone H3 and specific DNA repair gene loci. We conducted immunochemical and proteomic analyses to assess alterations in histone modification patterns. Transient effects were observed at both methylated and acetylated residues, with hypoacetylation specifically detected at promoters of certain DNA repair genes, including *MLH1*, *MSH2*, *MPG*, and *XPA*. Among all modifications analyzed, H3K18ac exhibited the most pronounced decline, suggesting its preferential sensitivity toward arsenite. H3 hypoacetylation was further observed in noncancerous human BEAS-2B lung cells, indicating that this effect is not cancer cell-specific. Mechanistically, in A549 cells, increased total HDAC or decreased HAT activity could be excluded. Instead, a persistent moderate decline in HDAC activity and a delayed, pronounced induction of HAT activity suggest targeted arsenite interactions with specific enzymes of the histone acetylation regulatory network.

## Graphical abstract

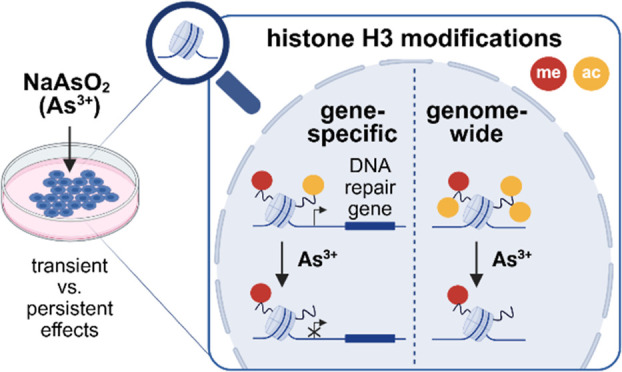

## Introduction

Arsenic is a naturally occurring metalloid found in soil, water, and the atmosphere, well-known for its carcinogenic properties.^1^ Extensive epidemiological research and studies in both in vitro and in vivo models have clearly linked exposure to inorganic arsenic with an increased risk of tumors in multiple organs, including skin, lung, bladder, kidney, and liver.^2^ Consequently, inorganic arsenic is classified as a human carcinogen.^3–5^ Even moderate dietary intake levels of arsenite, such as those found in Europe, are considered critical.^6^ The toxicologically most relevant inorganic arsenic species is trivalent arsenite, which exerts the highest carcinogenic potential due to its strong affinity for thiol groups in proteins, thereby disrupting key cellular processes.^7^ Unlike most carcinogens, arsenite itself is a weak mutagen.^8^ However, the mutagenic effect of distinct carcinogens can be potentiated by arsenite, most likely due to DNA repair inhibition.^9,10^ While inhibition at the protein level has been linked to direct interactions with zinc-binding motifs in DNA repair proteins,^11^ increasing attention has turned to arsenite-mediated epigenetic dysregulation.^12–14^ Specifically, histones, as key components of chromatin structure and transcriptional activity, may be particularly sensitive targets of arsenite-induced epigenetic alterations.^15–17^

Histones are small, positively charged proteins that form the structural units of chromatin, the nucleosomes.^18,19^ Negatively charged DNA is wrapped around histone octamers, which are composed of a tetramer of H3 and H4, along with two H2A and H2B dimers. These core histones enable the compact and organized packaging of DNA within the nucleus. Histones are thus pivotal in regulating gene expression by modulating chromatin organization, either through condensation or relaxation.^18^ Post-translational modifications (PTMs) on serine, arginine, and lysine residues, along with histone variants, contribute to a complex regulatory histone code.^18,20^ Among the most studied histone PTMs (hPTMs) are lysine acetylation and methylation. These are mediated by specific “writers” and “erasers”, such as histone acetyltransferases (HATs) and histone deacetylases (HDACs).^21,22^ Their effects depend on their location within genomic elements such as promoters or enhancers. Activating marks, e.g., H3K4me3, H3K9ac, and H3K18ac, are strongly associated with actively transcribed regions, while repressive hPTMs like H3K27me3 correlate with transcriptional inactivity.^18,21^

Arsenite is a well-documented inducer of chromosomal changes, primarily by altering hPTM processes, which in turn disrupt higher-order chromatin structures.^23,24^ Studies have shown its ability to affect some histone H3 acetylation and methylation sites on a global scale, likely via interference with histone-modifying enzymes.^16,25–27^ In addition, down-regulation of DNA repair genes has been linked to the accumulation or depletion of specific hPTMs at gene regulatory loci,^15,17^ suggesting a role in arsenite-mediated inhibition of DNA repair. However, most studies focused on individual hPTMs, using antibodies raised against selected marks, and on single DNA repair pathways, without addressing the persistence of these effects. This study aimed to fill these gaps by examining how hPTMs change under low-micromolar arsenite-induced cellular stress and after recovery, tracking the epigenetic response, while using biochemical and omics methods. We first investigated H3 PTM alterations in key DNA repair genes and integrated these with gene expression data. Moreover, we performed an antibody-based and unbiased proteomic analysis of bulk histone H3 PTMs to identify in both canonical H3 and variant H3.3 which lysine modifications are significantly affected by arsenite. These orthogonal approaches provided complementary and overlapping information in H3K9/K14ac, H3K18ac, and H3K36me2 variations, offering new insights into the impact of arsenite on histone H3 PTMs.

## Experimental Procedures

### Materials

All chemicals were obtained from Carl Roth (Karlsruhe, Germany) or Sigma-Aldrich (Massachusetts, USA). Cell culture media and materials were purchased from Sarstedt (Nuembrecht, Germany) and Lonza (Basel, Switzerland), with additives including fetal bovine serum from Thermo Fisher Scientific (Waltham, MA, USA) and Penicillin–Streptomycin from Sigma-Aldrich (Burlington, MA, USA). The ATP-Assay reagent was bought from Promega (Madison, WI, USA). FACSFlow and FACSRinse were supplied by BD (Franklin Lakes, NJ, USA), and DAPI (1 g/L) was purchased from Sigma-Aldrich (Burlington, MA, USA). PCR consumables were sourced from Brand (Wertheim, Germany) while PCR reagents were acquired from Applied Biosystems (Waltham, MA, USA), Bio-Rad (Munich, Germany), Fluidigm (San Francisco, USA) Machery-Nagel (Dueren, Germany), and Teknova (Hollister, USA). Primers were synthesized by Eurofins (Luxembourg City, Luxembourg). Antibodies and ChIP reagents were obtained from Cell Signaling Technology (Leiden, Netherlands) and Santa Cruz Biotechnology (Dallas, TX, USA). Histone extraction reagents for Western blot analysis were bought from Sigma-Aldrich (Burlington, MA, USA). LC–MS/MS consumables were purchased from Affinisep (Le Houlme, France) and Aurora (Collingwood, Australia). ELISA kits and nuclear isolation buffers were supplied by EpigenTek (Farmingdale, NY, USA).

### Antibodies

The hPTM and ChIP control antibodies were obtained from Cell Signaling Technology (Leiden, Netherlands) including H3K4me3 Tri-Methyl-Histone H3 (Lys4) (C42D8) Rabbit mAb no. 9751, H3K9ac Acetyl-Histone H3 (Lys9) (C5B11) Rabbit mAb no. 9649, H3K18ac Acetyl-Histone H3 (Lys18) (D8Z5H) Rabbit mAb no. 13998, and H3K27me3 Tri-Methyl-Histone H3 (Lys27) (C36B11) Rabbit mAb no. 9733, Histone H3 (D2B12) XP Rabbit mAb (ChIP Formulated) no. 4620, and Normal Rabbit IgG no. 2729. For Western blotting, the secondary antibodies were obtained from Santa Cruz Biotechnology (Dallas, TX, USA), including mouse antirabbit IgG-HRP no. 2357, m-IgG Fc BP-HRP no. 525409, and the loading controls β-Actin antibody (C4) no. 47778 and Histone H3 antibody (1G1) no. 517576.

### Cell Cultivation

A549 cells (ATCC CCL-185), a human lung adenocarcinoma cell line, were cultured in RPMI-1640 medium supplemented with 10% heat-inactivated fetal bovine serum, 100 U/mL penicillin, and 100 μg/mL streptomycin at 37 °C in a 5% CO_2_ and 100% humidified atmosphere. Passages 14–30 were utilized to ensure experimental consistency.

BEAS-2B cells (ATCC CRL-3588), a human noncancerous bronchial epithelial cell line, were cultured in KGM medium on flasks coated with fibronectin, collagen, and bovine serum albumin at 37 °C in a 5% CO_2_ and 100% humidified atmosphere. Passages 45–55 were used.

Cell identities and mycoplasma-free status were verified.

### Arsenite Treatment and Postincubation

Logarithmically growing A549 cells were treated with NaAsO_2_ at low micromolar concentrations for 24 h. Cytotoxicity (0.5–50 μM) and gene expression (1–25 μM) analyses guided the selection of 5–20 μM for the analysis of epigenetic changes. BEAS-2B cells were treated with 2.5–10 μM NaAsO_2_. For postincubation studies, treated cells were immediately reseeded at the same vessel-to-cell ratio and cultivated for an additional 48 h recovery period in arsenite-free medium. Concurrently, unexposed control cells were simultaneously cultured and passaged to account for any effects arising from continuous passaging.

### Cytotoxicity Assessment

ATP levels were analyzed using the CellTiter-Glo Luminescent Cell Viability Assay Kit (Promega GmbH, Walldorf, Germany) as previously described.^12^ Cell cultivation and incubation were carried out on 96-well plates, with 3 × 10^4^ cells seeded per well. Logarithmically growing cells were treated with NaAsO_2_. For postincubation studies, cells were initially treated on plates (4.5 × 10^6^ per dish) and subsequently seeded into 96-well plates as described above. ATP levels were normalized to untreated controls.

For determining the relative cell count, 4.5 × 10^6^ cells were seeded in plates (150 mm) and treated as stated above. The cells were collected in fresh medium, and cell numbers were measured using the CASY TT cell counter (OMNI Life Science, Bremen, Germany). Cell viability was normalized to untreated controls.

### Intracellular Arsenic Levels

The intracellular arsenic concentration was measured by graphit furnace-AAS (PinAAcle 900 T, PerkinElmer, Rodgau, Germany). Initially, 4.5 × 10^6^ cells were seeded and treated as stated above. Before pelleting, the cell count and cell volume were determined using the CASY TT cell counter (OMNI Life Science, Bremen, Germany). For sample preparation, the cells were dissolved in 500 μL of 30% H_2_O_2_ and 96% HNO_3_ (1:1, v/v) and heated stepwise to 95 °C. After complete evaporation of the solution, the remaining residue was dissolved in 0.2% HNO_3_. An external calibration was performed using AAS elemental standard solutions to ensure accurate measurements. The temperature program consisted of drying at 120 °C for 45 s and 140 °C for 30 s, pyrolysis at 1200 °C for 20 s, atomization at 2000 °C for 5 s, and tube cleaning at 2450 °C for 3°s.

### Analysis of Cell Cycle Distribution

The cell cycle analysis was conducted by flow cytometry (BD, Heidelberg, Germany) as previously described.^12,28,29^ For this study, 4.5 × 10^6^ cells were seeded and treated as described above, and about 2 × 10^6^ cells were fixed. The fluorescence signal was plotted against cell count to assess the distribution of cell cycle phases.

### Gene Expression Profiling

High-throughput gene expression profiling was performed as previously described.^12,28,30^ In this study, 5 × 10^5^ cells were seeded, and logarithmically growing cells were exposed to NaAsO_2_ and postincubated as described above. High-throughput RT-qPCR enabled simultaneous analysis of 96 samples for the expression of 95 genes using a 96 × 96 Dynamic Array integrated fluidic circuit system (Fluidigm, San Francisco, USA). Gene details are listed in Supporting Information Table S1, and primer sequences were published in.^12,30^ Data analysis was performed with Fluidigm Real-Time PCR Analysis and GenEx software (version 5.3.6.170). The reference genes *ACTB*, *B2M*, *GAPDH*, *GUSB*, and *HPRT1* were used for normalization. The relative quantities were calculated using the ΔΔ*C*q-method, with results expressed as log_2_-fold changes compared to respective controls.

### Gene-Specific Analysis of Histone Modifications

To detect hPTMs at specific DNA repair gene promoters, a ChIP assay coupled with qPCR detection was performed. For this analysis, 4.5 × 10^6^ cells were seeded and treated as stated before. The SimpleChIP Plus Sonication Chromatin IP Kit (Cell Signaling Technology, Leiden, Netherlands) was used. First, chromatin was cross-linked with 1% formaldehyde for 10 min, neutralized with glycine for 5 min. After washing twice, fixed cells were scraped into ice-cold PBS with protease inhibitor cocktail (PIC) and pelleted by centrifugation. Pellets were resuspended in ice-cold ChIP Sonication Cell Lysis Buffer with PIC, incubated on ice for 10 min, and centrifuged. This process was repeated, with the second step using the ChIP Sonication Nuclear Lysis Buffer with PIC. The lysates were sonicated for 2:30 min using a Branson Sonifier W-250D (Branson, Ultrasonics, Danbury, USA) at 10% energy input with 1 s pulses (on/off), while cooling on ice. Fragmentation efficiency was verified by running an aliquot of the fragmented lysates on a 2% agarose gel. Sonicated lysates were diluted (1:5 ratio) in ChIP-Buffer with PIC, and a 2% input was aliquoted for each sample. Specific hPTM antibodies (10 μL per 500 μL lysate), mock IgG antibody (2 μL), or ChIP control H3 antibody (10 μL) were added to the samples and incubated overnight at 4 °C. ChIP-Grade Protein G Magnetic Beads (30 μL) were added and incubated for 2 h at 4 °C. Chromatin was eluted with elution buffer for 30 min at 65 °C, reverse cross-linked with NaCl and Proteinase K. DNA was purified using spin columns. To improve DNA yield, a preamplification step and exonuclease digestion were included (according to gene expression profiling protocol). qPCR was performed on a CFX96 Touch Real-Time PCR Detection System (Bio-Rad, Feldkirchen, Germany) with Sso Fast EvaGreen with Low ROX (Bio-Rad, Feldkirchen, Germany) master mix using specific primers for DNA repair gene promoters, as well as positive and negative controls (*GAPDH*, *MB*, *MYOD1*, and *RPL30*). Primer sequences are listed in Supporting Information Table S3. The thermal protocol included an initial 180°s at 95 °C, followed by 40 cycles of 15 s at 96 °C (denaturation) and 60°s at 60 °C (annealing and elongation), followed by a melting curve analysis. ChIP-qPCR signals were calculated as a % of input.

### Global Analysis of Histone Modifications

Western blotting was used to analyze specific hPTMs genome-wide. A total of 4.5 × 10^6^ cells were seeded and treated as described above. Histones were extracted using the Core Histone Isolation Kit (Sigma-Aldrich, Taufkirchen, Germany) following the manufacturer’s instructions. Briefly, cells were lysed, the cytoplasmic fraction was separated, and histones were isolated from the nuclear fraction through acid extraction. The histone extracts were separated via SDS-PAGE and transferred to a PVDF membrane. Ponceau staining was performed as a loading control and documented using the LAS-3000 (FujiFilm, Tokyo, Japan). Proteins and antibodies were blocked with nonfat milk in 0.1% TBS-T. Primary and secondary antibody incubations were followed by chemiluminescent signal detection using Amersham ECL Western blotting detection reagent (GE Healthcare, UK), visualized with the LAS-3000 (FujiFilm, Tokyo, Japan) imaging system. In addition to detecting the respective hPTMs, β-Actin and histone H3 were also analyzed as loading controls. Before detecting H3, the membrane was stripped to prevent signal interference.

### Proteomic Analysis of H3 PTMs

For the proteomic analysis of hPTMs, 4.5 × 10^6^ cells were seeded and treated as stated before. Histones were acid-extracted and analyzed by LC–MS/MS as described elsewhere.^31^ Briefly, histones were derivatized using propionic anhydride in two rounds: pre- and post-trypsinization (at protein and peptide levels) at reactive free amines of unmodified and monomethylated lysines in addition to the free peptide N-termini. The undesired O-propionylation on serine, threonine, and tyrosine residues was reversed using hydroxylamine (0.5 M) with ammonium hydroxide (pH 12). The reaction was stopped by adding a few drops of pure TFA. The samples were then desalted using SPE tips (BioSPE PurePep) (Affinisep, Le Houlme, France) and vacuum-dried until later use. After derivatized histone peptides were resuspended in (5% ACN, 0.1% TFA), they were separated via a C18 column (C18, 25 cm × 75 μm, 1.7 μm beads, 120 Å pore size) (Aurora, Collingwood, Australia) of a liquid chromatography system (Vanquish Neo) then sprayed into an Ascend mass spectrometer (Thermo Fisher Scientific, Massachusetts, USA) that was operated in data-dependent acquisition (DDA) mode. Details of the LC gradient (Buffer A [0.1% FA in H_2_O]; Buffer B [ACN with 0.08% v/v FA]) and MS parameters are summarized in Supporting Information Tables S4 and S5, respectively.

For data analysis, the RAW files were converted into peak lists and searched by MASCOT software (Matrix Science v2.8) against an in-house curated histone database and contaminant database.^32^ The modifications were configured as follows: propionyl (Any N-term) set as a fixed modification, and propionyl (K), acetyl (K), butyryl (K) (standing for the sum of a methyl and a propionyl on the same lysine side chain), dimethyl (K), and trimethyl (K) set as variable modifications. Mass tolerance on peptides and fragments was set to 5 and 20 ppm, respectively. Identification result DAT files were imported into Proline software^33^ for identification validation (Mascot rank 1, cutoff score 30, minimum length of 6 amino acids) and label-free quantification by using the MS1 intensity at the chromatographic peak apex, and the match-between-runs option. The relative abundance (RA) of each sequence × PTM combination was calculated as follows

$$RA=\sum \frac{intensity\;of\;a\;\left(modified\right)\;sequence}{intensity\;of\;all\,\;modified\;sequence\;forms}$$

The data was visualized as bar plots representing the mean of 3 individual biological replicates that were shown as jittered points. The line segment represents the upper and lower limits of the confidence interval at 0.95%. Data analysis and visualization were carried out using in-house written scripts (https://github.com/HijaziHassan/histonePTM).

### Measurement of HDAC and HAT Enzymatic Activity

HDAC and HAT activity were measured using ELISA-based methods. A total of 4.5 × 10^6^ cells were treated with arsenite as described above. Nuclear extracts were prepared using the EpiQuick Nuclear Extraction Kit I (EpigenTek, New York, USA) according to the manufacturer’s instructions. To enhance extraction yield, all samples were further sonicated using a probe sonicator at 10% amplitude, with a 1 s on and 2 s off pulsed pattern for 30 s. For activity measurements, 5 μg of protein from nuclear extract was used. HDAC activity was determined with the Epigenase HDAC Activity/Inhibition Direct Assay Kit Colorimetric, and HAT activity was assessed using the EpiQuik HAT Activity/Inhibition Assay Kit, both from EpigenTek (New York, USA), following the manufacturer’s protocol. The enzymatic activities of HDAC and HAT in treated cells were calculated relative to those of unexposed control cells.

### Statistical Analysis

All cell culture experiments were conducted in duplicate across at least three independent experiments except for LC–MS/MS and ChIP-qPCR analyses. Statistical analyses were performed using one-way ANOVA followed by Dunnett’s post hoc test to evaluate the significance of treated cells relative to the control. For AAS, ANOVA with Šídák’s post hoc test was applied to compare 0 and 48 h postincubation. For high-throughput RT-qPCR analysis, two-way ANOVA followed by Dunnett’s post hoc test was performed across all genes to assess the significance of the exposed cells relative to the untreated control. For LC–MS/MS results, three biological replicates were analyzed in two independent experiments, with confidence intervals calculated. ChIP-qPCR was performed in single replicates across at least three independent experiments and analyzed statistically using Welch’s test. All statistical evaluations were performed using GraphPad Prism 10.4.0.621.

## Results

### Persistence of Arsenite-Induced Cellular Responses

Given that lung cancer is among the most sensitive end points of arsenic toxicity,^6^ we selected human lung cells as a model system. To assess the extent of arsenite-induced cellular stress and its persistence, we first examined cytotoxicity profiles of the human lung tumor cell line A549. Following previous reports of our group,^12,29^ A549 cells were exposed to NaAsO_2_ for 24 h at initial doses up to 50 μM. For the postincubation study, the cells were subsequently cultured in arsenite-free medium for an additional 48 h. First, intracellular ATP levels, as a marker of cytotoxicity, were determined to identify suitable exposure conditions. Based on these results, concentrations of 1–20 μM arsenite were selected for subsequent experiments. This range reflects environmentally and occupationally high exposure acute scenarios and was chosen to remain below general cytotoxicity in vitro. In the general European population, blood arsenic levels are typically around 30 nM, rising to approximately 300 nM in occupationally exposed individuals^34^ and up to 9 μM in populations residing in highly contaminated regions such as parts of India or Bangladesh.^35^ In these regions, groundwater arsenic levels frequently exceed the WHO limit of 10 μg/L. In some areas, these levels reach up to approximately 27 μM, which is around 200-fold above the WHO limit.^36^ To confirm that the selected concentrations triggered adaptive and stress-related rather than nonspecific cytotoxic responses, we further analyzed the relative cell count, intracellular arsenic content using atomic absorption spectroscopy (AAS), and cell cycle phase distribution via flow cytometry. At the transcriptional level, high-throughput RT-qPCR was performed to examine various cellular stress response genes, including those involved in (oxidative) stress response, inflammation, cell cycle regulation, DNA repair and damage response, as well as epigenetic regulation.

### Postincubated Cells Exhibited No Signs of Cytotoxicity Recovery after 48 h

Figure 1A/B presents the cytotoxic potential of arsenite in A549 cells, assessed first by measuring ATP content. ATP levels serve as an indicator of cellular metabolic activity and viability, and measurements were performed both without and with a 48 h arsenite-free recovery period. Within the 0.5 μM to 15 μM range, no signs of cytotoxicity were observed. However, starting from 20 μM, a moderate decrease in ATP was detected, though the levels remained above 80% of control at 20 μM and 25 μM arsenite. At 50 μM, ATP levels dropped to approximately 60% in both setups. Thus, postincubation did not further affect cytotoxicity, nor did it allow restoration of intracellular ATP.

**Figure 1:**
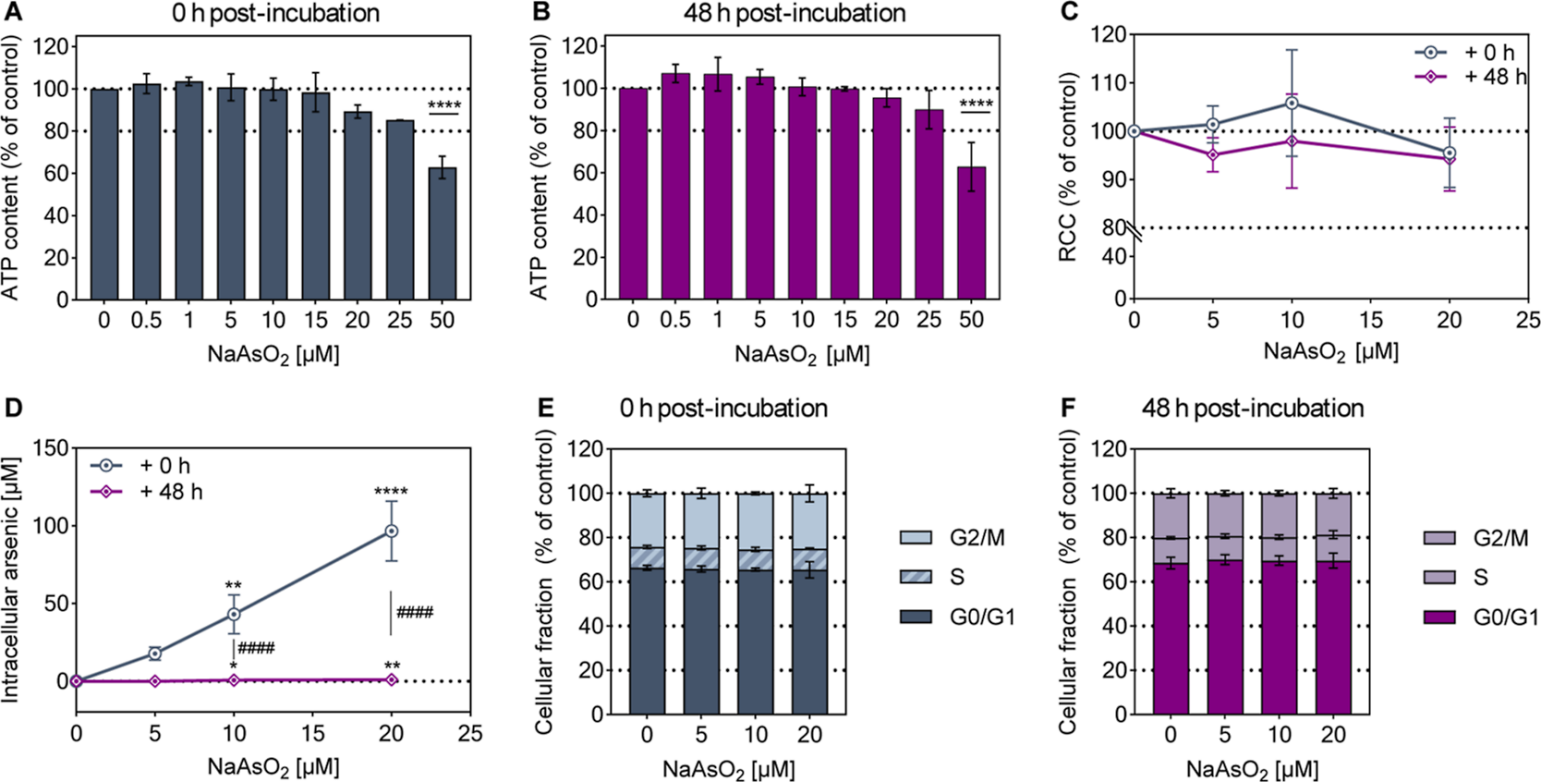
Assessment of arsenite cytotoxicity, uptake, and impact on cell cycle progression in A549 cells. Cells were treated with NaAsO_2_ for 24 h, followed by either no (0 h) or a 48 h postincubation period in arsenite-free cell culture medium. ATP content was analyzed immediately after arsenite treatment (A) or 48 h afterward (B). The relative cell count (RCC) was assessed using CASY cell count analysis (C), and the cellular uptake of arsenic was quantified by AAS (D). Moreover, the cell cycle distribution was analyzed by flow cytometry using DAPI staining (E,F). Shown are mean values ± SD from three independent experiments, each conducted in duplicate. Statistical analysis was performed using a one-way ANOVA followed by a Dunnett’s post hoc test to evaluate which of the observed changes in exposed cells reached statistical significance compared to the untreated control: *­(*p* < 0.05), **­(*p* < 0.01), ****­(*p* < 0.0001). To assess the effect of postincubation, statistical significance was determined using ANOVA with Šídák’s post hoc test: #### (*p* < 0.0001).

The cell count analysis within this dose range also showed no changes (Figure 1C). The values remained near 100%, both with and without postincubation.

To explore whether this outcome was influenced by intracellular accumulation of the metalloid, total arsenic levels were measured at both time points (Figure 1D). Subsequently, after treatment, a dose-dependent increase in intracellular arsenic was observed, reaching nearly 97 μM per cell at 20 μM NaAsO_2_. Notably, following the postincubation, the levels decreased significantly. At 5 μM, levels were barely measurable. At 10 μM and 20 μM, residual nanomolar doses were detected. These correspond to a reduction to approximately 1.2% and 0.9%, respectively. This pronounced decline indicates efficient cellular excretion within this time frame. Additionally, cell cycle analysis revealed no phase changes within the selected dose range, irrespective of postincubation (Figure 1E/*F*).

### Postincubated Cells Show More Pronounced Changes in the Expression of Specific Genes

To analyze the time-dependent transcriptional response of A549 cells to NaAsO_2_ (1–25 μM), gene expression profiles were assessed for 95 cellular stress-related genes using high-throughput RT-qPCR.^12,30^ Exploratory analysis revealed significant differences in gene activity between solely arsenite-treated and additionally 48 h postincubated cells. Figure 2 represents a volcano plot of the relative gene expression changes (RGE) in A549 cells treated with the highest concentration of 25 μM NaAsO_2_. Figure 3 shows a heatmap of the RGE for selected genes categorized into functional clusters. Comprehensive log_2_-values for the gene panel and selected RGE plots are provided in Supporting Information Table S2 and Figure S1.

**Figure 2:**
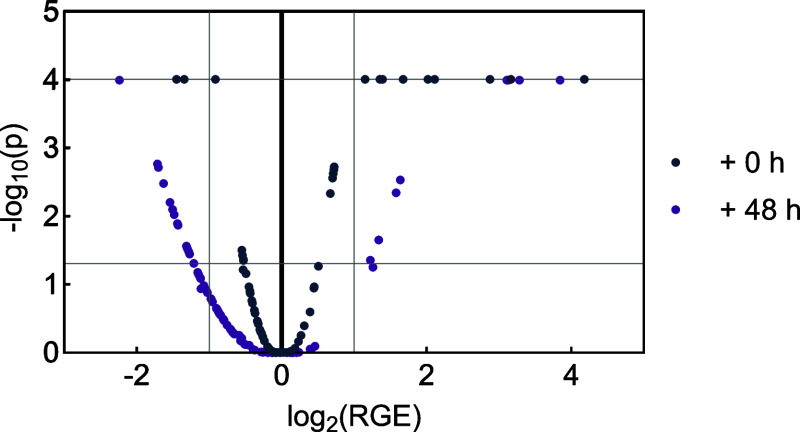
Effect of arsenite and postincubation on the gene expression profile of A549 cells. Cells were exposed to 25 μM NaAsO_2_ for 24 h and subsequently subjected to either a 0 or 48 h arsenite-free recovery phase. The mRNA levels were assessed by high-throughput RT-qPCR in 95 genes. Displayed is the relative change in gene expression (RGE) compared to the untreated control against the p-value generated by two-way ANOVA, followed by Dunnett’s post hoc test. For simplification purposes, all *p*-values <0.0001 are displayed on the line of *p* = 0.0001.

**Figure 3:**
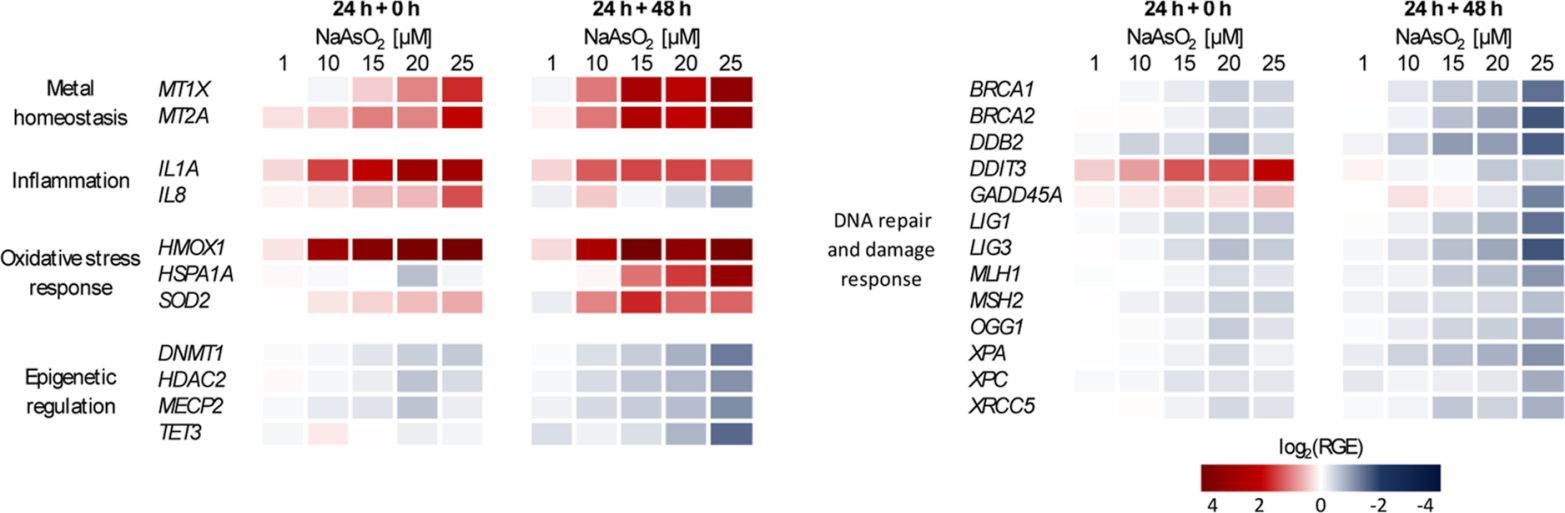
Gene expression profiles of arsenite-treated and postincubated A549 cells. Cells were exposed to NaAsO_2_ in the dose range of 1 μM to 25 μM for 24 h and subsequently subjected to either a 0 or 48 h arsenite-free recovery period. Gene expression was determined using high-throughput RT-qPCR. The results are presented as log_2_-fold change of the relative gene expression (RGE). Gene induction is represented in red color; gene repression is depicted in blue. Shown are mean values from at least three independent experiments, each conducted in duplicate.

Considering the distribution of the data points, including and excluding postincubation, a trend toward effect intensification was observed with extended postincubation time (Figure 2). This is evidenced by a broader distribution of data, reflected in a widening parabolic shape with additional recovery time. Furthermore, the number of relevant gene repressions increased, as shown by a higher density of data points surpassing *p* < 0.005 and exhibiting gene expression changes of <1, corresponding to at least a 50% reduction compared to untreated controls.

Postincubation of acutely arsenite-treated A549 cells resulted in distinct changes in the mRNA levels. In the following, the results for selected genes shown in Figure 3 are discussed. Notably, significant changes in RGE were observed primarily starting at 10 μM NaAsO_2_, yet trends were already emerging at 1 μM.

Specifically, genes related to metal homeostasis, such as *MT1X* and *MT2A*, displayed higher induction following a 48 h recovery phase. At the highest dose of 25 μM, postincubation led to a 10-fold increase in *MT1X* transcript levels and a 9-fold increase for *MT2A*, compared to 3-fold and 4-fold increases, respectively, observed immediately after NaAsO_2_ treatment.

Furthermore, arsenite induced a persistent inflammatory response, primarily driven by the sustained induction of *IL1A*. However, *IL8* expression levels returned to basal states.

The most pronounced RGE alterations following arsenite treatment were observed in oxidative stress response genes. HMOX1 exhibited the most significant induction of all genes. Starting at 10 μM, a 24 h incubation resulted in an 8-fold increase in transcript levels, which exceeded an 18-fold increase at the highest dose. Even after the postincubation, induction remained substantial, showing a 6-fold and 14.5-fold increase at 25 μM. Interestingly, acute NaAsO_2_ treatment did not significantly alter mRNA levels of *HSPA1A* or *SOD2*. However, after the 48 h recovery phase, these genes were up-regulated, with *HSAP1A* increasing 9-fold and *SOD2* doubling.

In addition, arsenite exposure induced DNA damage markers, with levels returning to baseline 48 h post-treatment, potentially indicating the completion or discontinuation of DNA repair processes. For instance, *DDIT3* levels were up-regulated directly after treatment, but returned nearly to the basal state after 48 h. Overall, most DNA repair factors examined demonstrated a repressive transcriptional behavior after acute arsenite exposure. This includes genes involved in various repair pathways: *BRCA1*, *BRCA2* (homologous recombination), *DDB2* (nucleotide excision repair (NER)), *OGG1*, *LIG1*, and *LIG3* (base excision repair (BER)), as well as *MLH1* and *MSH2* (mismatch repair (MMR)). The repression of these genes was intensified after the additional postincubation period. For instance, basal transcript levels of most genes declined further, except for *MSH2*, where the reduced levels remained stable. At the highest dose of 25 μM NaAsO_2_, the extent of repression differed between targets, with some genes showing more pronounced suppression (e.g., *MLH1* or *BRCA2*) than others (e.g., *OGG1*). Accordingly, all DNA repair genes except *OGG1* showed varying degrees of reduction to below 50% of their original transcript levels. Such variability in inhibition strength likely reflects gene-specific promoter accessibility due to epigenetic alterations.

In addition, epigenetic regulatory genes were also assessed. Following 24 h acute arsenite exposure, an overall decline in transcriptional levels of epigenetic regulators was observed, with some showing even increased repression after the postincubation period. Transcripts of *DNMT1*, *HDAC2*, and *MECP2* were repressed by arsenite, with reduction exceeding 50% after recovery at the highest dose. The repression of *TET3* was particularly detectable 48 h after treatment at the highest concentrations (15–25 μM).

### Epigenetic Insights into H3 Modification Changes Induced by Arsenite

We investigated hPTM patterns of histone H3, focusing also on the effect persistence. Considering alterations in gene expression and cytotoxicity results, low micromolar concentrations of 5 μM to 20 μM were selected for epigenetic analysis. We initially conducted postincubation studies to investigate gene-specific responses of selected DNA repair genes and their associations with hPTMs, analyzed through chromatin immunoprecipitation (ChIP) coupled with quantitative PCR (qPCR). Subsequently, global assessments of the selected gene-specific hPTMs were carried out using Western blotting, providing a broader perspective on arsenite-induced hPTM alterations. Finally, to identify additional arsenite-sensitive hPTM patterns, these findings were complemented by comprehensive hPTM landscape profiling via liquid chromatography coupled with tandem mass spectrometry (LC–MS/MS).

### Temporary Changes in H3 Modifications at Various DNA Repair Genes Caused by Arsenite

Acute exposure of A549 cells to low micromolar concentrations of NaAsO_2_ resulted in a sustained, moderate transcriptional repression of all DNA repair factors investigated (see Figure 3). To determine whether corresponding alterations in hPTMs occur at the promoters of selected DNA repair genes, we investigated the levels of specific gene-activating marks (H3K4me3, H3K18ac, H3K9ac) and one gene-repressive mark (H3K27me3). Our study expanded upon earlier research, which primarily focused on single DNA repair pathways and individual modifications,^15,17^ by examining multiple hPTMs at the promoter loci of *MPG* and *XRCC1* (BER), *MLH1* and *MSH2* (MMR), *XPA* and *XPC* (NER), as well as at positive and negative control loci. The selection of these modifications was designed to capture a broad spectrum of hPTMs, encompassing both transcriptional activating and repressive marks. The selected DNA repair genes have all been previously reported to be repressed by arsenite.^15–17,37^ The results of this analysis are presented in Figure 4. Additional controls, including an example of the fragment size distribution of chromatin fragments generated by sonication, are provided in Supporting Information Figures S2 and S3.

**Figure 4:**
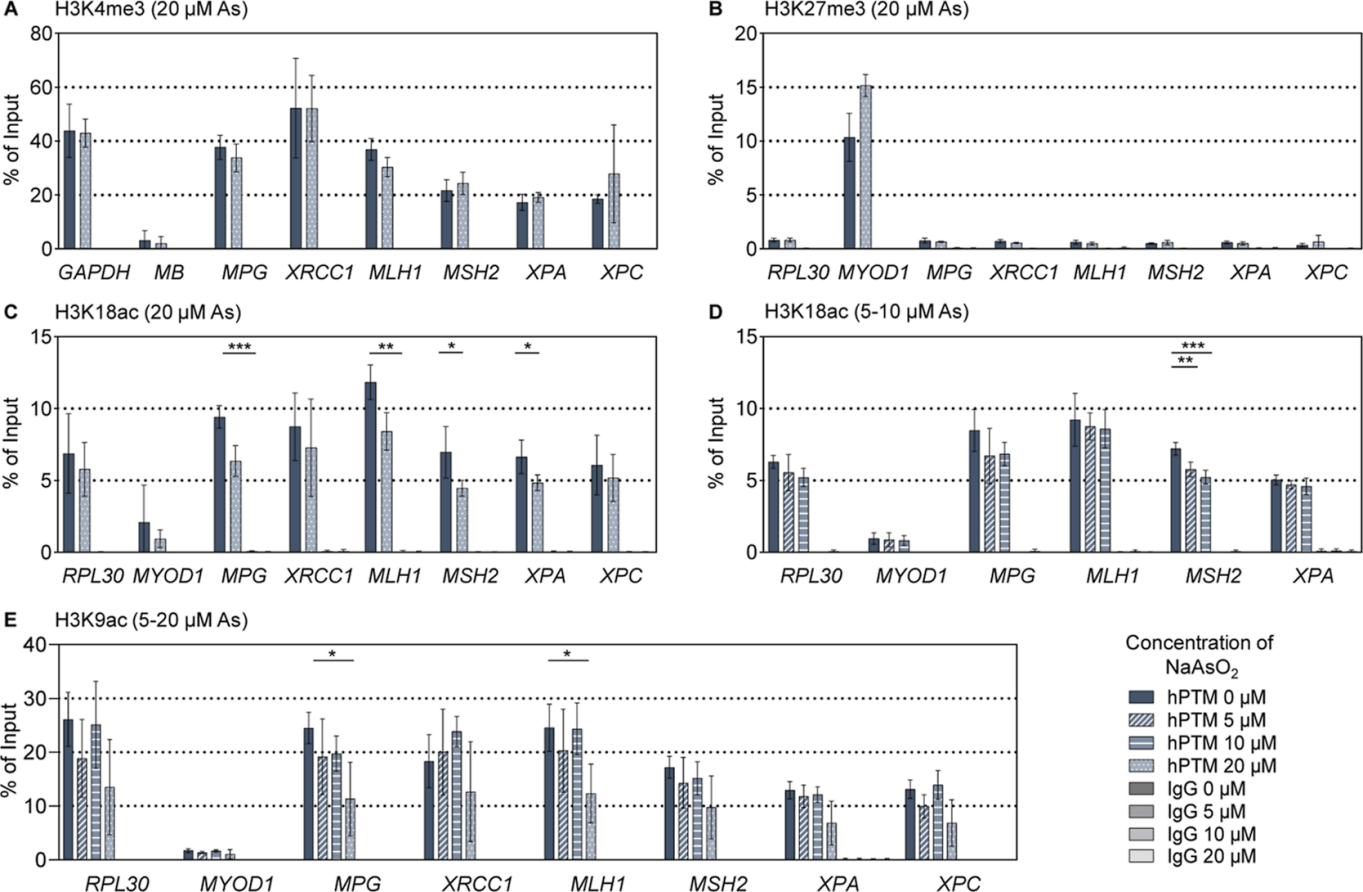
Basal versus arsenite-modified hPTM levels at selected DNA repair genes. A549 cells were incubated with different concentrations from 5 μM to 20 μM NaAsO_2_ for 24 h. (A) and (B) show the ChIP-qPCR results for H3K4me3 and H3K27me3 at 20 μM. (C) Shows the results for H3K18ac at 20 μM and (D) at 5 μM and 10 μM. (E) Displays the ChIP-qPCR data of H3K9ac in the whole dose range. As positive and negative control locus *RPL30*, *GAPDH*, *MB*, and *MYOD1* were determined. In addition, an IgG isotype control antibody was used to detect the nonspecific background. The corresponding ChIP controls for the enrichment of histone H3 at the positive control locus RPL30 can be found in the Supporting Information Figure S2. A representative example of the fragmentation pattern is given in Supporting Information Figure S3. Shown are the mean values ± SD of at least three independent experiments. Statistical analysis was performed using Welch’s test to evaluate which of the observed changes in exposed cells reached statistical significance compared to the untreated control: *­(*p* < 0.05), **­(*p* < 0.01), ***­(*p* < 0.001).

In the A549 cell line, the active histone mark H3K4me3 was substantially detected across all DNA repair-related promoters investigated, indicating active transcription of these genes (Figure 4A). Acute exposure to 20 μM NaAsO_2_ resulted in no significant changes in H3K4me3 abundance in the selected promoter regions.

Regarding the repressive histone mark H3K27me3, A549 cells displayed minimal signal at the selected promoter sites, further supporting the transcriptionally active states of these genes (Figure 4B). Exposure to 20 μM NaAsO_2_ did not result in any significant alterations in H3K27me3 levels. Consistently, enrichment levels remained below those observed at the *RPL30* negative control locus, with no discernible trends.

Since neither H3K4me3 nor H3K27me3 elicited any changes, further analyses with lower concentrations or postincubation were not pursued for these histone marks.

Moreover, a strong signal of the gene activating mark H3K18ac was detected at the respective promoter sites (Figure 4C/D). A 24 h treatment of A549 cells to 20 μM NaAsO_2_ led to a significant decline in H3K18ac levels at the promoters of *MPG*, *MLH1*, *MSH2*, and *XPA* (Figure 4C). In detail, H3K18ac levels decreased by 33% at *MPG*, 29% at *MLH1*, 36% at *MSH2*, and 27% at *XPA*, respectively. Further investigation of lower doses revealed a consistent down-regulation of H3K18ac at the *MSH2* promoter in response to arsenite (Figure 4D). Both 5 μM and 10 μM NaAsO_2_ exposure elicited reduced H3K18ac levels at *MSH2*, with a reduction by 20% (5 μM) and 27% (10 μM). This pattern aligns with our previous transcriptional findings that indicated a down-regulation of *MSH2* expression. To determine whether the H3K18ac levels exhibit sustained changes, an additional arsenite-free recovery period was included (see Supporting Information, Figure S4). However, no persistent effects were observed after the postincubation, suggesting transient changes of H3K18ac at specific DNA repair-related promoters.

Consistent with H3K18ac, also a strong signal for the hPTM H3K9ac was detected at the investigated promoter sites (Figure 4E). Cells treated with 5 μM to 20 μM NaAsO_2_ showed a decreasing trend in H3K9ac levels at nearly all tested loci at the highest dose. Significant H3K9ac declines of 54% and 50% were observed at *MPG* and *MLH1* promoters after arsenite exposure. However, it is important to note that the positive control locus *RPL30* also exhibited a slight decreasing trend at 20 μM NaAsO_2_, suggesting possibly a more generalized effect rather than a gene-specific response. As the changes were only evident at the highest dose and no differences were detected in initial postincubation experiments (data not shown), further loci-specific investigations for H3K9ac were not conducted.

### Dynamic Global Response of H3 Lysine Modifications to Arsenite Treatment

To gain insight into the persistence of the arsenite-induced bulk hPTM effects, the selected hPTMs were further analyzed by Western blotting. The relative global abundances of the modifications are depicted in Figure 5, with an example blot provided in Supporting Information Figure S5.

**Figure 5:**
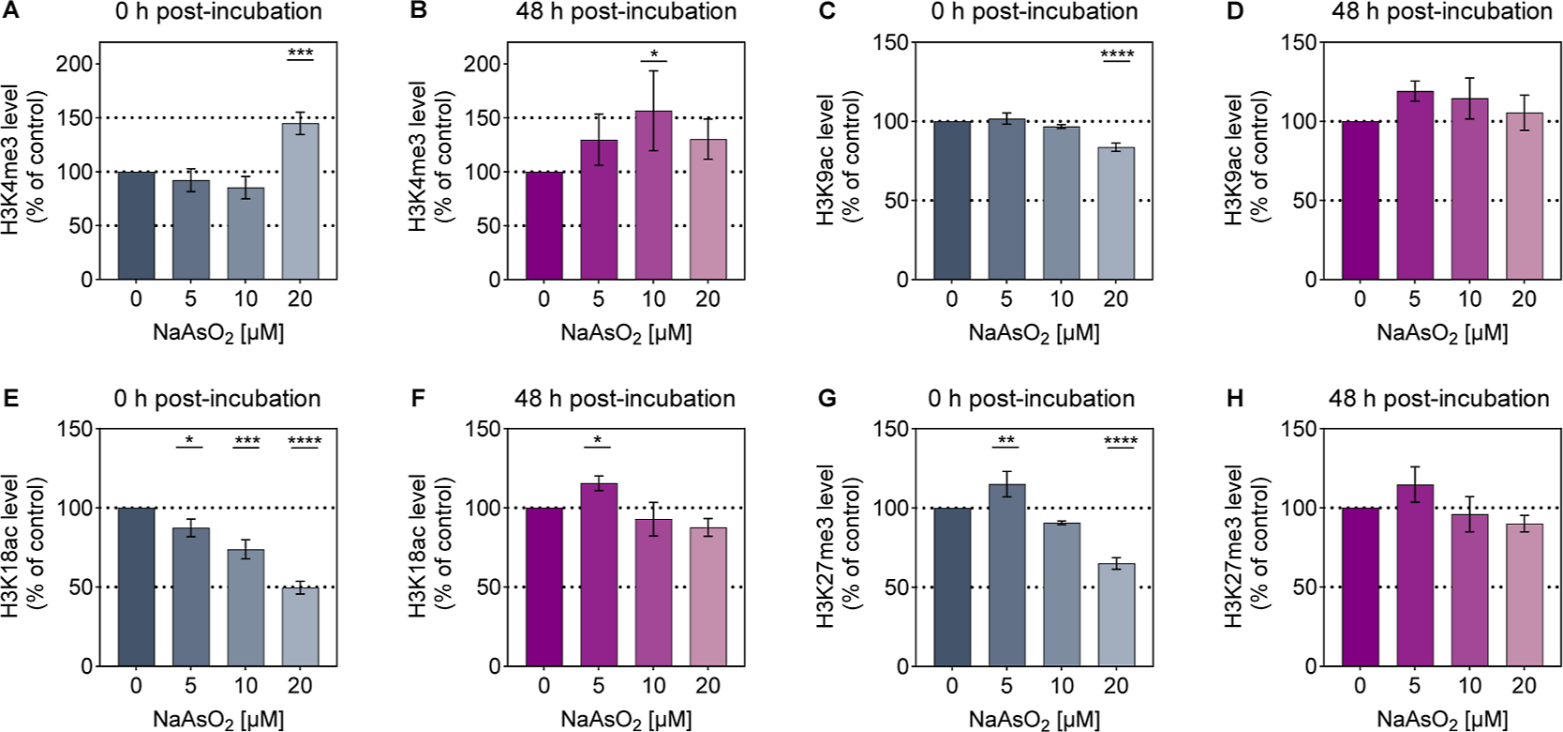
Effects of arsenite exposure and postincubation on the levels of various post-translational histone modifications in A549 cells. Cells were exposed to NaAsO_2_ for 24 h and subsequently subjected to either 0 h (A,C,E,G) or 48 h (B,D,F,H) arsenite-free recovery period. Relative modification abundance was detected using Western blotting. For the semiquantitative analysis presented here, the results were normalized to the respective loading controls (Ponceau, histone H3, and β-Actin) and averaged. Shown are mean values ± SD from at least three independent experiments. Statistical analysis was performed using a one-way ANOVA followed by a Dunnett’s post hoc test to evaluate which of the observed changes in exposed cells reached statistical significance compared to the untreated control: *­(*p* < 0.05), **­(*p* < 0.01), ***­(*p* < 0.001), ****­(*p* < 0.0001).

A 24 h treatment of A549 cells with 20 μM NaAsO_2_ resulted in a notable increase in H3K4me3 abundance of 45% compared to the control (Figure 5A). At lower concentrations (5 μM and 10 μM), no significant effects were detected, although a minor downward trend was apparent. An additional 48 h arsenite-free postincubation phase revealed dynamic changes in H3K4me3 levels (Figure 5B). While all arsenite concentrations exhibited an upward trend, a significant 57% increase was observed specifically at 10 μM. At 5 μM and 20 μM, H3K4me3 abundance rose by 1.3-fold. The elevated abundance at 20 μM persisted after postincubation, albeit with reduced intensity. These findings suggest a dynamic, time-dependent response in H3K4me3 levels following acute arsenite exposure, with evidence of persistent effects at 20 μM.

Regarding H3K9ac, the acute treatment of the cells with NaAsO_2_ resulted in a moderate decrease of H3K9ac at the highest dose, with no significant changes observed at lower doses (Figure 5C). Specifically, exposure to 20 μM NaAsO_2_ led to a 16% decline in H3K9ac abundance compared to the basal state. After an additional arsenite-free 48 h postincubation, H3K9ac levels at 20 μM recovered to baseline, while a slight upward trend was noted at lower doses (Figure 5D). These findings align with the gene-specific results, indicating that arsenite induces a marginal, transient reduction in H3K9ac at the highest dose, which is fully restored following the recovery phase.

In the case of H3K18ac, arsenite exhibited a pronounced dose-dependent decline in H3K18ac abundance in A549 cells (Figure 5E). Starting from the lowest concentration tested, H3K18ac levels decreased significantly. At 5 μM, a decline to 87% of basal levels was observed. At 10 μM, 74% remained. The strongest reduction was observed at the highest dose of 20 μM, with H3K18ac levels reaching only 50% of control. Following a 48 h postincubation period without arsenite, H3K18ac levels showed partial recovery (Figure 5F). At 5 μM, even a slight yet significant 1.2-fold increase was observed. However, at 10 μM and 20 μM, minor downward trends were noted. Altogether, the most pronounced changes occurred immediately after the 24 h NaAsO_2_ treatment, with H3K18ac levels reduced to approximately 50% of control levels. Subsequent recovery nearly restored H3K18ac levels with a marginal increase at 5 μM. Thus, similar to the gene-specific observations, low-micromolar arsenite exposure resulted in transient hypoacetylation within the tested dose range.

Arsenite also induced significant changes in H3K27me3 levels, displaying a dose-dependent pattern of both increases and decreases (Figure 5G). At 5 μM, a slight but significant increase of 15% compared to the control was detected. At 20 μM, a significant decline of 35% was observed. After the two-day recovery phase, H3K27me3 levels at 10 μM and 20 μM nearly returned to baseline (Figure 5H). At 5 μM, the increasing trend persisted. These results indicate that arsenite induces transient changes in H3K27me3 expression at 10 μM and 20 μM, while at 5 μM, levels remain stable and slightly elevated.

Given that arsenite exposure leads to hypoacetylation of H3K9 and H3K18 in A549 lung tumor cells, we investigated whether noncancerous lung cells exhibit the same epigenetic response. Therefore, we assessed H3K9ac and H3K18ac levels in the nonmalignant lung cell line BEAS-2B. Due to a higher arsenite sensitivity of BEAS-2B compared to A549 cells (data not shown), the dosage was adjusted to 2.5–10 μM NaAsO_2_. The results are shown in Figure 6, with a representative blot provided in Supplementary Figure S6.

**Figure 6:**
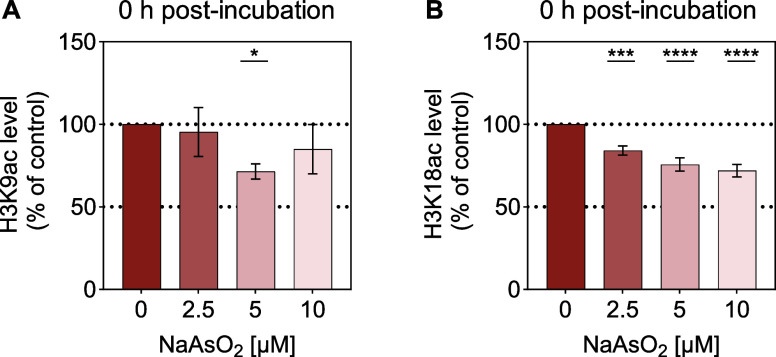
Effects of acute arsenite exposure on the level of H3K9ac and H3K18ac in BEAS-2B cells. PTM levels were detected using Western blotting. The values were normalized to the respective loading controls (Ponceau, histone H3, and β-Actin) and averaged. Shown are mean values ± SD from at least three independent experiments performed. Statistical analysis was performed using a one-way ANOVA followed by a Dunnett’s post hoc test to evaluate which of the observed changes in exposed cells reached statistical significance compared to the untreated control: *­(*p* < 0.05), *** (*p* < 0.001), **** (*p* < 0.0001).

In the case of H3K9, a significant hypoacetylation was detected at 5 μM (Figure 6A). Arsenite led to a 29% decline in H3K9ac levels compared to the basal state. At 10 μM, the hypoacetylation was less pronounced, which may be attributed to the beginning of cytotoxic effects at this dose.

Moreover, H3K18 displayed a concentration-dependent hypoacetylation starting from 2.5 μM. The relative acetylation levels decreased from 84% (2.5 μM) down to 76% (5 μM) and 72% (10 μM). Similar to A549 cells, arsenite caused H3K9 and H3K18 hypoacetylation in BEAS-2B cells. These findings suggest that hypoacetylation may be a regulatory mechanism in the cellular response to arsenite in lung-derived cells.

### Arsenite Alters the Landscape of Histone H3 PTMs

To assess the impact of arsenite on histone modifications more exhaustively, we conducted a comparative proteo-mic analysis of the H3 PTM landscape using histone ex-tracts derived from arsenite-treated versus control A549 cells. Our analysis focused specifically on identifying modifications in lysine residues of histone H3. Briefly, histones were propionylated at their free lysines and proteolyzed with trypsin, to produce peptides all ending with arginine and containing between one and three lysines. Samples were analyzed by LC–MS/MS to quantify the relative abundance of each modified form of a given peptide sequence. Figure 7 shows peptides with modified acetylation and methylation states. Additional profiling results are provided in Supporting Information Figure S7.

**Figure 7:**
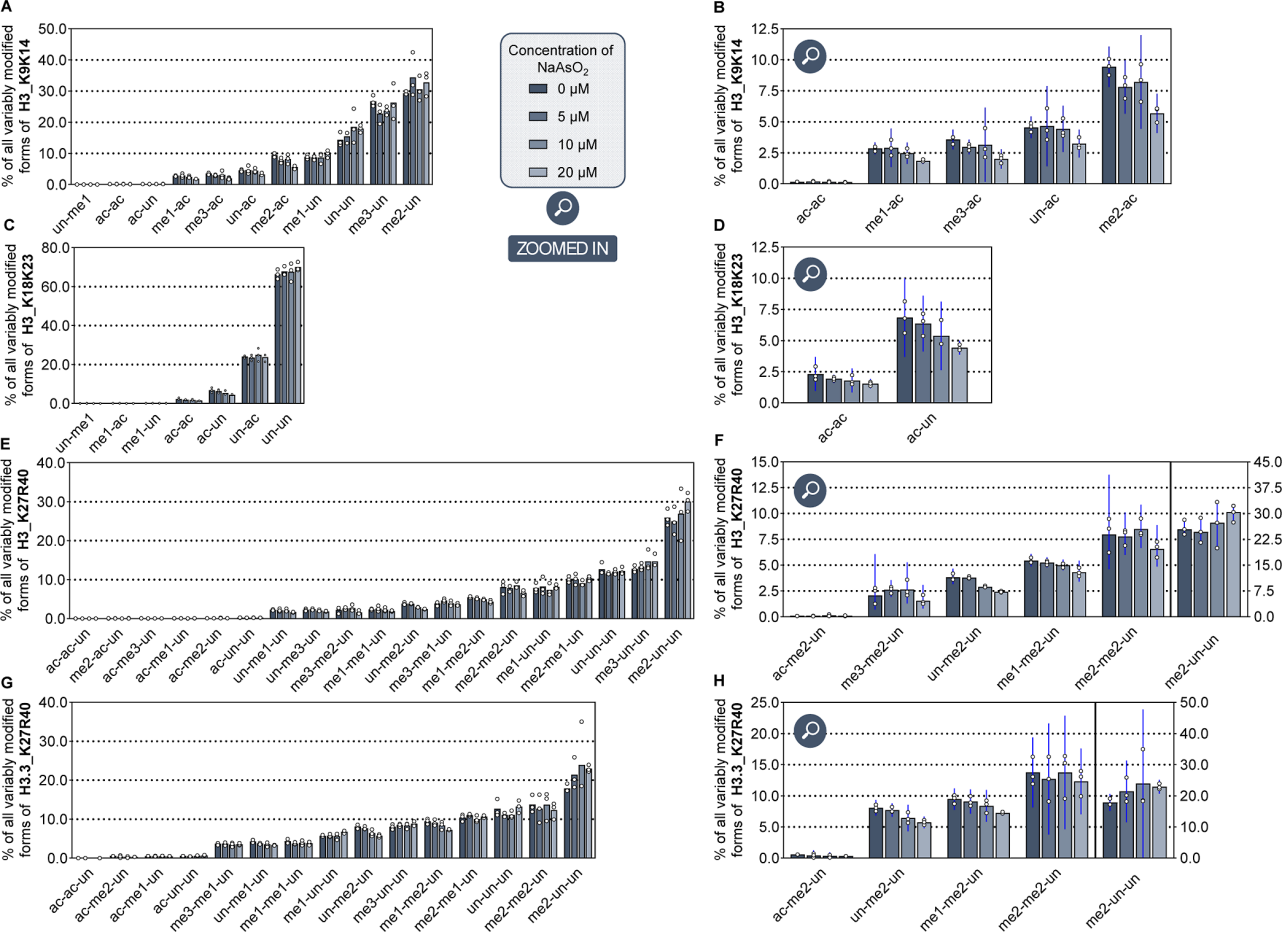
Relative abundances of the variably modified forms of selected peptides from histone H3 upon treatment of A549 cells with increasing arsenite concentrations. The cells were incubated with 5 μM to 20 μM NaAsO_2_ for 24 h. Post-translational histone modifications were analyzed by LC–MS/MS. The data are shown for all modified forms of peptide spanning residues K9-R17 (A), K18-R26 (C), K27-R40 from canonical H3 (E) and variant H3.3 (G), and the respective zoomed in versions show PTM combinations at K9/K14 (B), K18/K23 (D), and K27/K36 (F,H) of these sequences significantly changing in abundance. The quantitative data obtained from biological triplicates were plotted as individual dots. The bar heights correspond to the mean ± confidence interval at 95% confidence level. The histograms of the other analyzed peptides from H3 are represented in Supporting Information Figure S7.

Overall, specific alterations were observed, suggesting that arsenite induces targeted regulatory mechanisms. Starting at the N-terminus of H3, we detected a consistently low abundance of H3K9ac that remained largely unaffected by arsenite, while a clear trend toward hypoacetylation was noted for H3K14ac, interestingly observed in combination with H3K9me1, H3K9me2, and H3K9me3 (Figure 7A/B). This decreasing trend extended to H3K18ac, whereas H3K23ac levels remained stable (Figure 7C/D). In contrast, lysine residues further downstream of the N-terminus displayed changing patterns in their methylation states. Notably, our analysis distinguished between the K27-R40 peptide from H3.1 (canonical H3) and that from variant H3.3, which differs by an Ala/Ser switch at position 31. The most pronounced effects were observed for K36me2 in both H3.1 and H3.3, starting at 10 μM NaAsO_2_. Interestingly, this trend was observed in combination with H3K27unmod and H3K27me1 (7E-H). Conversely, we observed an increase by about 30% in H3.3K27me2 in combination with K36unmod at the highest arsenite concentration compared to controls (Figure 7H). Overall, these analyses provided a refined characterization of PTM combinations at K9/K14, K18/K23, and K27/K36 from histone H3, some of which exhibited a significant change upon arsenite. The selective change for only some of these PTM patterns likely reflects changes at specific genomic regions.

To complete the analysis of H3 PTMs, we hypothesized a potential difference in the abundance of H3.1 versus H3.3 in response to arsenite exposure. However, this hypothesis was excluded by comparing the summed MS signals corresponding to variably modified K27-R40 peptide sequences from H3.1 and H3.3 (see Supporting Information, Figure S8). Moreover, we also tested for direct arsenic binding to histone H3 cysteines but found no peptides with matching theoretical mass.

### Prolonged Impact of Arsenite on Writers and Erasers of Histone Acetylation

To explore the cause of deacetylation, we examined the activity of histone acetylation regulators. These include also cysteine-containing writers and erasers,^38–40^ making them particularly sensitive to arsenite.^41^ An ELISA-based approach was applied to measure the total activity.

### Arsenite Leads to a Persistent and Modest Reduction of HDAC Activity

To gain a better understanding of how arsenite affects the histone acetylation status, total nuclear HDAC activity was assessed. A 24 h treatment of A549 cells with NaAsO_2_ resulted in a moderate, dose-dependent reduction in HDAC enzymatic activity (Figure 8A). While no statistically significant effects were observed at 5 μM and 10 μM, a significant decrease down to 86% of the control was detected in nuclear extracts from cells treated with 20 μM NaAsO_2_. After a subsequent 48 h recovery period, the reduced HDAC activity at 20 μM persisted at 86% of control (Figure 8B). Additionally, treatment of the nuclear extracts with the HDAC inhibitor Trichostatin A (TSA) revealed a significant decrease in HDAC activity, both without and with the recovery period (Figure 8C/D). TSA alone caused a comparable reduction of HDAC activity down to nearly 68% of control under both conditions. When TSA treatment was combined with arsenite-exposed extracts, no clear intensification of the inhibitory effect was observed. At 20 μM, arsenite-treated extracts showed a slight additional reduction, reaching approximately 66% and 59% of control at both time points, respectively. This indicates that arsenite does not markedly amplify TSA-induced HDAC inhibition in this experimental setup. It is important to note that the applied method was limited to detecting class I and class II HDACs (HDACs 1–11). This limitation may exclude potential contributions from the class III sirtuin (SIRT) family to the observed effects.

**Figure 8:**
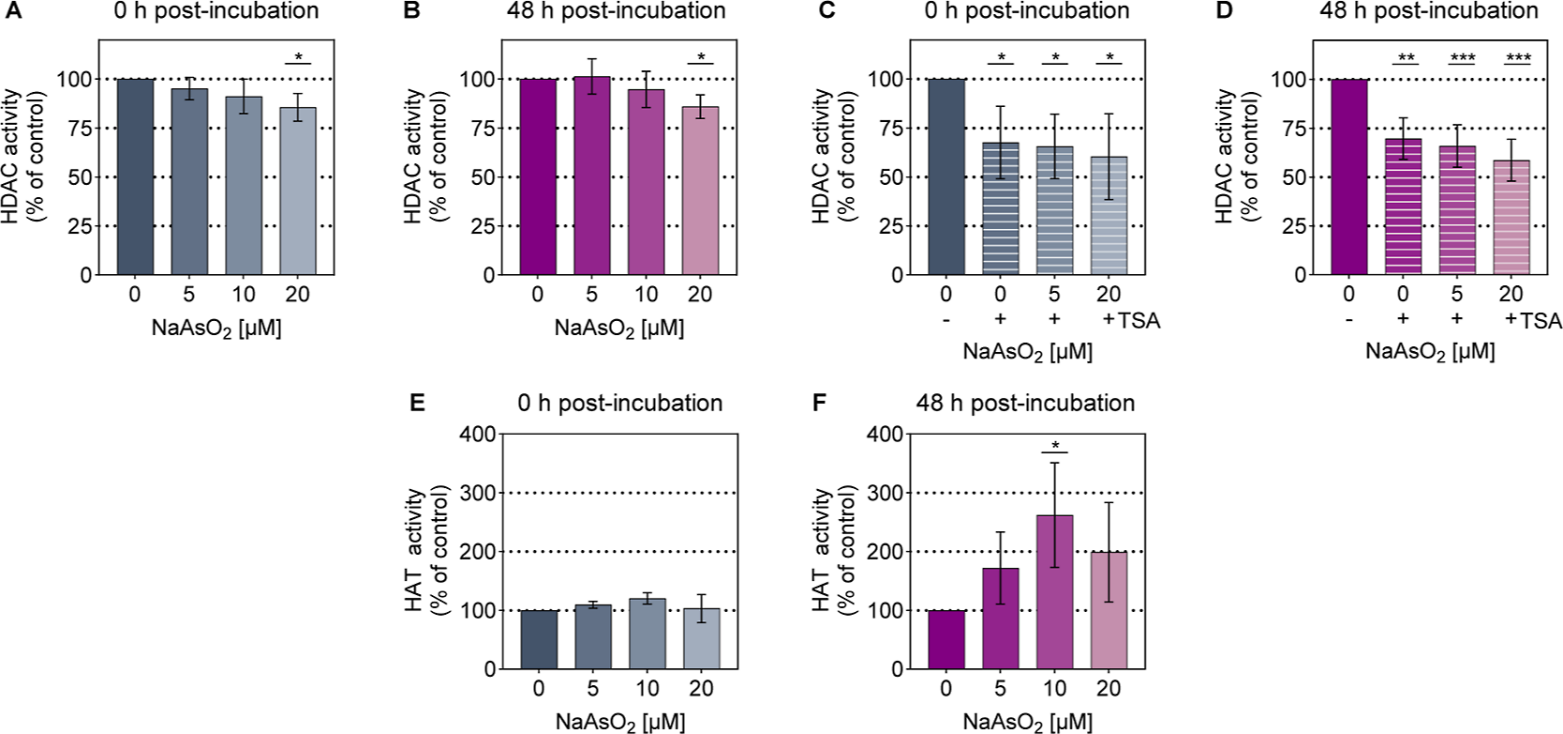
Effect of arsenite exposure and postincubation on the total HDAC and HAT activity in A549 cells. Cells were exposed to NaAsO_2_ for 24 h and subsequently subjected to either 0 h (A,C,E) or 48 h (B,D,F) recultivation in the absence of arsenite. HDAC and HAT activity were determined by ELISA-based methods. The HDAC activity is shown in (A,B), while (C,D) display the results with an additional 2 μM Trichostatin A (TSA) HDAC inhibitor treatment of the nuclear extracts. The HAT activity is displayed in (E,F). Shown are mean values ± standard deviation from at least three independent experiments performed in duplicate. Statistical analysis was performed using a one-way ANOVA followed by a Dunnett’s post hoc test to evaluate which of the observed changes in exposed cells reached statistical significance compared to the untreated control: *­(*p* < 0.05), **­(*p* < 0.01), *** (*p* < 0.001).

### Arsenite Modulates HAT Activity with a Delayed Enhancement Post-Recovery

Given that the equilibrium between acetylating and deacetylating enzymes is crucial, the activity of HATs was additionally examined. The arsenite-exposed A549 nuclear extracts initially exhibited a slight, increasing trend in HAT activity, particularly at 10 μM (Figure 8E). Following a 48 h arsenite-free recovery period, the nuclear extracts showed a strong induction in HAT enzymatic activity (Figure 8F). The most pronounced increase occurred at 10 μM NaAsO_2_, with a 162% rise. At 5 μM and 20 μM, increasing trends of 72% and 99% were detected. Notably, the 10 μM arsenite-treated extracts demonstrated a significant induction in enzymatic activity, a phenomenon that emerged only after the 48 h recovery period. These findings suggest a delayed but pronounced response in HAT activity, particularly at 10 μM arsenite, underscoring the dynamic interplay between acetylating and deacetylating enzymes in response to arsenite exposure.

## Discussion

Arsenite, a known carcinogen, disrupts DNA repair through protein interference, but the mechanisms driving transcriptional repression of DNA repair genes remain unclear. This study supports a role for altered hPTMs in mediating this repression, yielding new perspectives into arsenite-mediated epigenetic dysregulation.

### Arsenite Induces Persistent Transcriptional Changes

Here, we demonstrate that even a single low-micromolar exposure to arsenite can cause persistent transcriptional repression in human cells, particularly affecting DNA repair and epigenetic regulators. Notably, despite the almost complete drop of intracellular arsenic levels during the postincubation period, gene-specific repression not only persisted but even intensified after the two-day recovery period. This decline was likely due to efflux via ABC transporters, cell division dilution, and passive diffusion through uptake channels.^42^ These findings suggest that arsenite may remain tightly bound to specific cellular targets, thereby creating a lasting cellular memory, potentially driven by epigenetic mechanisms known to maintain altered gene expression through reversible modifications.^43^ Although the residual intracellular levels declined to less than 1% of the initial amount, resulting in levels of 0.5–1 μM, they appear to be sufficient for the observed effect persistence. The prolonged repression coincided with sustained ATP depletion, indicating impaired cellular recovery. Nevertheless, the continued gene-specific transcriptional dysregulation of stress-response genes implies sustained interactions with critical molecular targets. Supporting this notion, chronic arsenite exposure studies have shown similar long-term transcriptional dysregulation.^44^ Moreover, our study demonstrates that even acute exposure (24 h) can induce sustained disruptions in transcriptional regulation of various genes, highlighting the ability of arsenite to trigger lasting epigenetic changes. Previous work in HaCaT cells supports this notion, although limited to DNA methylation regulators and MMR genes.^45^

### Arsenite Mediates Transient Histone Hypoacetylation at Different DNA Repair Genes

Epigenetic alterations play a pivotal role in cellular responses to environmental stress. Among these, the fine-tuned network of chemical modifications on histones regulating the chromatin structure and accessibility is highly sensitive to carcinogenic agents such as arsenite.^23,24^ These hPTMs can exert targeted regulatory effects, leading to the down-regulation or activation of specific genes. Given that the arsenite-induced transcriptional changes persisted after the recovery period and showed an intensification across the DNA repair gene set (resembling basically all major DNA repair pathways), we subsequently analyzed specific genes and associated hPTMs. Our findings provide supporting evidence that arsenite mediates transient hypoacetylation of specific histone residues at the promoter regions of various DNA repair genes. In particular, H3K18ac showed significant hypoacetylation at the promoters of genes involved in BER (*MPG*), MMR (*MLH1*, *MSH2*), and NER (*XPA*). The most pronounced decline was observed for *MSH2*, independently of the respective dose, in agreement with its observed transcriptional down-regulation. However, this hypoacetylation dissipated after a two-day recovery period, suggesting that the changes were transient. In addition, H3K9ac was hypoacetylated at the promoters of *MPG* and *MLH1* at the highest dose. This pattern aligns with previous studies in HaCaT cells, which demonstrated that the repressive mark H3K9me2 increased at *MPG*, *XRCC1*, and *PARP1*,^15^ and that H3K18ac decreased at the promoter loci of *XPA*, *XPD*, and *XPF*.^17^ These findings may partially explain the described inhibition of the corresponding DNA repair pathways. Notably, the present study is the first to demonstrate that arsenite induces hypoacetylation of specific histone residues at MMR gene promoters. Among all investigated pathways, MMR was the most affected in our experimental setup. Similar disruption of DNA repair pathways, particularly MMR, and resulting microsatellite instability have already been identified as critical events in metal-induced tumorigenesis, as shown for chromium.^46,47^ MMR is crucial for correcting replication errors and preserving genomic stability. However, its downregulation may act as a pro-survival mechanism under stress, promoting cellular resistance.^48,49^ Previous reports have shown that arsenite induces DNA hypermethylation in *MLH1* and *MSH2* promoters, leading to gene silencing.^16^ In HaCaT cells, *MSH2* appears particularly sensitive, as its promoter remained hypermethylated even after a single acute arsenite exposure.^45^ This aligns with our finding that the *MSH2* promoter is particularly susceptible to arsenite-induced epigenetic reprogramming. Consequently, *MSH2* may constitute a key epigenetic target of arsenite.

It is important to note that the hypoacetylation of DNA repair genes was transient, whereas transcriptional repression persisted (e.g., *MLH1* or *MSH2*). Thus, the sustained reduced mRNA levels cannot be attributed to H3 hypoacetylation alone but likely involve epigenetic crosstalk. Here, the initial hypoacetylation of histone H3 may serve as a priming event, inducing a repressive chromatin environment. This could subsequently trigger other epigenetic mechanisms such as DNA methylation at specific gene loci or chromatin remodeling processes, and ncRNA expressions, ultimately resulting in sustained transcriptional repression.^18,50,51^ Although it remains unclear whether hPTMs are drivers or consequences of genomic regulation, a recent study suggests they could function as both.^18^

### Arsenite Triggers Dynamic Global H3 PTM Changes

Consistent with the gene-specific findings, our analysis of selected marks of bulk histones confirms that hPTMs are highly dynamic and temporally responsive to environmental stress. Notable changes were observed in H3K4me3, H3K27me3, H3K9ac, and H3K18ac levels during the recovery phase, with H3K18ac showing the strongest effects. The detected transient global decline of acetylation levels of H3K9 and H3K18 aligns with the observed gene-specific hypoacetylation of these lysines and suggests that hPTMs contribute to finely tuned regulatory cell responses. Importantly, hypoacetylation occurred in both cancerous and noncancerous human lung cells, showing that the effect is not restricted to tumor cells.

While previous work has examined H3K4me3 dynamics after subchronic arsenite exposure,^52^ our study is, to our knowledge, the first to characterize global hPTM changes following recovery from acute arsenite exposure. Together, these findings suggest that exposure duration critically influences the persistence of hPTM alterations.

To complete the investigation by Western blot of selected hPTMs, the spectrum of global H3 modification changes induced by a 24 h arsenite treatment was explored without a priori using a proteomic approach. Our first aim was to identify additional hPTMs potentially modulated by arsenite and to compare these findings with the antibody-based results. In detail, we identified new molecular targets, specifically decreased acetylation at H3K14, and increased dimethylation at H3K36 in both canonical H3 and variant H3.3. Very interestingly, the increase in H3K36me2 was detected when combined with unmodified, acetylated, and monomethylated H3K27, while the decrease in H3K14ac co-occurred with mono-, di-, and trimethylated H3K9. This likely indicates a change in H3K36me2 (especially in variant H3.3) and H3K14ac levels at various genomic regions. However, we could not detect a significant decrease of H3K9ac. As H3K14ac is much more abundant than H3K9ac, it may happen that the signal detected by Western blot using an antibody raised against H3K9ac actually comes from H3K14ac. Besides, our proteomic analysis would reveal increased H3K27me2 in combination with unmodified H3K36 and K37, in particular in variant H3.3, whereas it did not confirm changes in H3K27me3. The proteomics data also confirmed H3K18 hypoacetylation, in good agreement with the Western blot results. Finally, our proteomic method could not detect the very small peptide containing H3K4me3, which is known to be very hydrophilic and usually overlooked, unless an alternative derivatization protocol is applied.^53^ These observations underscore the importance of integrating multiple analytical methods to comprehensively capture the full spectrum of arsenite-induced hPTM targets.

Indeed, reported inconsistencies in the literature, e.g., for H3K9 acetylation, for instance, range from hyperacetylation^26^ to hypoacetylation^54,55^ or no significant changes.^56^ Similar discrepancies exist for other marks, including H3K4me3, H3K18ac, and H3K27me3, as previously reviewed.^57^ These inconsistencies may be related to differences in experimental systems and methodologies, including the limited specificity of antibodies, with respect to the sequence (e.g., possible cross-reactivity between close lysine sites) and to the PTM (e.g., cross-reactivity between di- and trimethylation), as probably exemplified by our findings. In general, factors such as exposure duration, arsenite concentration, and the use of male- or female-derived cells may also contribute to the variability.^58^

### Arsenite Disrupts HDAC/HAT Balance

To investigate whether arsenite-induced hypoacetylation involves altered corresponding enzyme activities, we measured total HDAC and HAT activity. Given the critical importance of the balance between these two antagonistic enzyme classes,^59^ our results provide evidence that arsenite disrupts this equilibrium persistently. Overall HDAC activity decreased both immediately after exposure and following the recovery phase, whereas total HAT activity significantly increased during the subsequent 2 d postincubation period. This suggests a pivotal role for HATs in cellular restoration and survival processes after arsenite exposure. However, these results do not support the hypothesis that the acute genome-wide and gene-specific hypoacetylation arises from reduced HAT or increased HDAC activity. Instead, the data point toward the opposite effect, indicating a potential hyperacetylation response.

Studies in HepG2 cells identified arsenite as a potent HDAC suppressor, comparable to the pan-HDAC inhibitor TSA.^26^ Additionally, a recent study showed that arsenite suppresses HDACs at both the transcriptional and protein levels in rats.^60^ In our gene expression analysis, we assessed various *HDACs* but did not detect notable transcriptional changes immediately after treatment, with *HDAC2* decreasing solely after 48 h postincubation.

Our data demonstrate that acute arsenite exposure of A549 cells induces locus-specific histone hypoacetylation, independent of global HDAC or HAT activity changes, suggesting a targeted mechanism. Such specificity may involve direct inhibition of individual regulators, as seen for PARP-1,^61^ cysteine-rich HATs such as p300/CBP,^62^ and hMOF.^40^ Arsenite has also been shown to down-regulate class III HDACs, the sirtuins (SIRTs). Specifically, previous studies reported that arsenite triggers *miR-34a* expression, which destabilizes *SIRT1* mRNA and leads to its down-regulation, partly through aberrant DNA methylation.^63–65^ Gene-specific effects of SIRTs, such as SIRT7 activity at ELK4-regulated promoters,^66^ further support this hypothesis. Additionally, transcription factors like HIF-1α, a known repressor of *MSH2* and an arsenite-inducible factor,^67–69^ may interact with histone-modifying enzymes to mediate these effects. Moreover, gene-specific interactions with corepressors such as TRIM33 ^70^ may also lead to gene-specific hypoacetylation. In addition, reduced acetylation levels could also result from protein degradation, as arsenite has been shown to target the zinc-binding motif of HAT TIP60, leading to its ubiquitin-proteasome-mediated degradation.^71^ Furthermore, reduced cellular acetyl-CoA availability may contribute to the observed hypoacetylation.^72^

Overall, this study uncovers novel aspects of epigenetic regulation in response to acute arsenite-induced cellular stress. Our experimental design comprises both low- and high-dose acute exposure scenarios (1–25 μM) to capture a broad range of stress responses. While this design provides valuable mechanistic insights, future studies using lower concentrations and conducting long-term studies will help to understand whether these findings are also relevant for environmental exposure levels in noncontaminated areas. While our findings primarily focused on histone acetylation, the analyses also highlighted the involvement of histone methylation in arsenite-induced hPTM changes. Particularly, the methylation of H3K36, whose dimethylated state was identified to be significantly increased by arsenite, may be linked to arsenite-induced inhibition of the MMR system, a connection previously demonstrated for the trimethylated state H3K36me3.^16^ Future studies should focus on specific arsenite–enzyme interactions, including writers, erasers, and readers of hPTMs—a topic currently under investigation by our group. Analyzing the precise binding of these enzymes to substantial gene loci could provide critical insights into the targeted effects of arsenite on chromatin structure and gene expression. Importantly, a comprehensive understanding of the hPTM landscape, including the regulatory network encompassing diverse modifications, histone variants, and associated proteins, is essential. This includes deciphering the interplay between hPTMs and other epigenetic mechanisms, such as DNA methylation, chromatin remodeling and the impact of noncoding RNA expression. Such knowledge will be pivotal for advancing our understanding of arsenite-induced interactions and pathogenesis under low-exposure conditions.

## Conclusions

In this study, to the best of our knowledge, we investigated for the first time the impact of acute arsenite exposure followed by an arsenite-free recovery phase on the cellular stress response and on the global and DNA repair-related acetylation and methylation of histone H3. The data demonstrate that a single acute exposure of human A549 lung tumor cells to arsenite leads to persistent repression of DNA repair genes and epigenetic regulators up to 48 h postexposure, suggesting epigenetic transcriptional dysregulation. These effects occurred at noncytotoxic concentrations, comprising low to high acute exposure conditions. Significant H3K18 hypoacetylation was observed at the promoters of *MPG*, *MLH1*, *MSH2*, and *XPA* at 20 μM NaAsO_2_. The most pronounced decline was already detectable at 5 μM for *MSH2*, correlating with its transcriptional down-regulation. However, this effect was abolished 48 h postexposure, suggesting transient hPTM changes. Additionally, H3K9 hypoacetylation was also detected at the *MPG* and *MLH1* promoters at 20 μM. These gene-specific findings are consistent with our global analysis showing transient hypoacetylation. However, sustained transcriptional repression could not be fully explained, potentially pointing to crosstalk with DNA methylation and other epigenetic processes. These results obtained by classical biochemical methods were supported and completed by the proteomic analysis of H3, which confirmed the decrease of H3K18ac, indicated a decrease of H3K14ac, and highlighted an increase of H3K36me2 upon arsenite treatment. Importantly, this hypoacetylation occurred without corresponding changes in total HAT or HDAC activity, suggesting specific epigenetic targeting rather than global enzymatic inhibition. Still, HAT and HDAC activities remained altered beyond 48 h, alongside transcriptional changes in stress-response genes, indicating the presence of arsenite-sensitive targets. Additionally, noncancerous BEAS-2B lung cells likewise showed H3K9 and H3K18 hypoacetylation, confirming that the effect is not specific to cancer cells. These findings reveal a complex regulatory network and emphasize the need for further investigation of mechanisms driving arsenite-induced epigenetic changes.

## Supplementary Material



## Notes

Additional experimental details for the HT-RT-qPCR and ChIP-qPCR analyses are provided in Tables S1–S3 and Figures S1–S4; Representative immunoblots are shown in Figure S5 and S6; Additional LC–MS/MS results and method details are presented in Tables S4 and S5 and Figures S7 and S8 (PDF)

## Abbreviations

AAS: atomic absorption spectroscopy
BER: base excision repair
ChIP: chromatin immunoprecipitation
HAT: histone acetyltransferase
HDAC: histone deacetylase
hPTM: histone post-translational modification
LC-MS/MS: liquid chromatography coupled with tandem mass spectrometry
MMR: mismatch repair
NER: nucleotide excision repair
RGE: relative changes in gene expression
SIRT: sirtuin
TSA: Trichostatin A.
