# Supplementary material for: Impact of Arsenite on Transient and Persistent Histone H3 Modifications and Transcriptional Response
Source: Chem Res Toxicol. 2026 Jan 2;39(1):64–78. doi: 10.1021/acs.chemrestox.5c00312 (PMC12820973; doi:10.1021/acs.chemrestox.5c00312)
Supplement: Supplementary file 1 [file tx5c00312_si_001.pdf]

Supplementary information to:

Impact of Arsenite on Transient and Persistent Histone H3 Modifications and Transcriptional Response

Tatjana Lumpp †, Hassan Hijazi ‡, Sandra Stößer †, Eda Tekin †, Lara Brunner †, Franziska Fischer †, Sabine Brugière ‡, Delphine Pflieger ‡, and Andrea Hartwig †*.

† Karlsruhe Institute of Technology, Institute of Applied Biosciences, Department of Food Chemistry and Toxicology, Adenauerring 20a, 76131 Karlsruhe, Germany

‡ University Grenoble Alpes, INSERM, CEA, UA13 BGE, CNRS, CEA, FR2048, Grenoble, France

Supplementary Table S1: List of all analyzed genes and their respective proteins examined via high throughput RT-qPCR. Reference genes are indicated by an asterisk (*).

Gene	Encoded protein
*ACTB**	β-actin (ACTB)
APAF1	apoptotic protease activating factor (APAF1)
ATM	ataxia telangiectasia mutated (ATM)
ATR	ataxia telangiectasia and Rad3-related protein (ATR)
*B2M**	beta-2-Microglobulin (B2M)
BAX	bcl2-associated x protein (BAX)
BRCA1	breast cancer 1, early onset (BRCA1)
BRCA2	breast cancer 2, early onset (BRCA2)
BTRC	transducin repeat containing E3 ubiquitin protein ligase, beta (β-TrCP)
CAT	catalase (CAT)
CCL22	C-C motif chemokine ligand 22 (CCl22)
CCND1	cyclin D1 (CCND1)
CDKN1A	cyclin-dependent kinase inhibitor 1A (p21)
CDKN1B	cyclin-dependent kinase inhibitor 1B (p27)
CDKN2A	cyclin-dependent kinase inhibitor 2A (p16)
CDKN2B	cyclin-dependent kinase inhibitor 2B (p15)
CDKN2D	cyclin-dependent kinase inhibitor 2D (p19)
COX2	cytochrome C oxidase assembly factor (COX2)
DDB2	damage-specific DNA binding protein 2 (DDB2)
DDIT3	DNA damage inducible transcript 3 (DDIT3)
DNMT1	DNA methyltransferase 1 (DNMT1)
DNMT3A	DNA methyltransferase 3A (DNMT3A)
DNMT3B	DNA methyltransferase 3B (DNMT3B)
E2F1	E2F transcription factor 1 (E2F1)
EGFR	epidermal growth factor receptor (EGFR)
EHMT2/G9a	euchromatic histone lysine methyltransferase 2(EHMT2)
EP300	E1A binding protein P300 (EP300)
ERCC2	excision repair cross-complementation group 2 (XPD)
ERCC4	excision repair cross-complementation group 4 (XPF)
ERCC5	excision repair cross-complementation group 5 (XPG)
FOXO1	forkhead box O1 (FOXO1)
FOXO3	forkhead box O3 (FOXO3)
FTH1	ferritin heavy chain 1 (FTH1)
G6PD	glucose-6-phosphate dehydrogenase (G6PD)
GADD45A	growth arrest and DNA-damage-inducible, alpha (GADD45A)
*GAPDH**	glyceraldehyde-3-phosphate dehydrogenase (GAPDH)
GCLC	glutamate-cysteine ligase, catalytic subunit (GCL)
GPX1	glutathione peroxidase 1 (GPX1)
GPX2	glutathione peroxidase 2 (GPX2)
GSR	glutathione reductase (GSR)
*GUSB**	glucuronidase, beta (GUSB)
HDAC1	histone deacetylase 1 (HDAC1)
HDAC10	histone deacetylase 10 (HDAC10)
HDAC2	histone deacetylase 2 (HDAC2)

HDAC3	histone deacetylase 3 (HDAC3)
HMOX1	heme oxygenase (decycling) 1 (hMO-1)
*HPRT1**	hypoxanthine phosphoribosyltransferase 1 (HPRT1)
HSPA1A	heat shock 70kDa protein 1A (HSP70)
IL1a	interleukin 1 alpha (IL1a)
IL1b	interleukin 1 beta (IL1b)
IL6	interleukin 6 (IL6)
IL8	interleukin 8 (IL8)
JUN	jun proto-oncogene (c-JUN)
KDM3A	lysine demethylase 3A (KDM3A)
KEAP1	kelch-like ECH-associated protein 1 (KEAP1)
LIG1	ligase I, DNA, ATP-dependent (LIG1)
LIG3	ligase III, DNA, ATP-dependent (LIG3)
MAP3K5	mitogen-activated protein kinase kinase kinase 5 (MAP3K5/ASK1)
MBD4	methyl-CpG binding domain 4 (MBD4)
MDM2	MDM2 proto-oncogene (MDM2)
MeCP2	methyl-CpG binding protein 2 (MeCP2)
MGMT	O-6-methylguanine-DNA methyltransferase (MGMT)
MLH1	mutL homolog 1 (MLH1)
MSH2	mutS homolog 2 (MSH2)
MT1X	metallothionein 1X (MT1X)
MT2A	metallothionein 2A (MT2A)
MYC	v-myc avian myelocytomatosis viral oncogene homolog (c-MYC)
NFKB1	nuclear factor of kappa light polypeptide gene enhancer in B-cells 1 (p50/p105)
NFKB2	nuclear factor of kappa light polypeptide gene enhancer in B-cells 2 (p49/p100)
NFKBIA	nuclear factor of kappa light polypeptide gene enhancer in B-cells inhibitor,alpha (IKBA)
OGG1	8-oxoguanine DNA glycosylase (hOGG1)
PARP1	poly (ADP-ribose) polymerase 1 (PARP1)
PLK3	polo-like kinase 3 (PLK3)
PMAIP1	phorbol-12-myristate-13-acetate-induced protein 1 (NOXA)
PRDX1	peroxiredoxin 1 (PRX1)
RAD50	RAD50 homolog (S. cerevisiae) (RAD50)
RAD51	RAD51 recombinase (RAD51)
RRM2B	ribonucleotide reductase M2B (TP53 inducible) (p53R2)
SETD2	SET domain containing 2 (SETD2)
SIRT2	sirtuin 2 (SIRT2)
SLC30A1	solute carrier family 30 (zinc transporter), member 1 (ZnT1)
SOD1	superoxide dismutase 1, soluble (SOD1)
SOD2	superoxide dismutase 2, mitochondrial (SOD2/MnSOD)
TET1	tet methylcytosine dioxygenase 1 (TET1)
TET2	tet methylcytosine dioxygenase 2 (TET2)
TET3	tet methylcytosine dioxygenase 3 (TET3)
TGFb	transforming growth factor beta (TGFb)
TNFa	tumor necrosis factor a (TNFa)
TNFRSF10B	tumor necrosis factor receptor superfamily, member 10b (DR5)
TXN	thioredoxin (TXN)
TXNRD1	thioredoxin reductase 1 (TXNRD)
VEGFA	vascular endothelial growth factor A (VEGFA)
XPA	xeroderma pigmentosum, complementation group A (XPA)
XPC	xeroderma pigmentosum, complementation group C (XPC)
XRCC5	x-ray repair complementing defective repair in Chinese hamster cells 5 (XRCC5)

Supplementary Table S2: Precise high-throughput RT-qPCR results. Relative gene expression is displayed as a log_2-fold change.

		24 h + 0 h					24 h + 48 h				
NaAsO2 [µM]		1	10	15	20	25	1	10	15	20	25
Metal homeo-stasis	*MT1X*	0.01	-0.10	0.39	0.98	1.68	-0.10	1.06	2.69	2.23	3.29
	MT2A	0.25	0.41	1.02	0.98	2.02	0.12	1.07	2.46	2.05	3.12
Inflam-mation	*IL1a*	0.33	1.48	2.13	2.92	2.88	0.35	1.29	1.46	1.47	1.34
	IL1b	-0.10	-0.06	0.35	0.87	1.15	-0.15	0.36	0.71	0.78	1.26
	IL6	0.14	1.58	2.43	2.70	3.17	-0.33	2.33	2.83	2.01	1.58
	IL8	0.10	0.19	0.55	0.59	1.39	-0.17	0.44	-0.09	-0.37	-1.02
Oxidative stress response	*GCLC*	-0.07	-0.36	-0.61	-0.93	-0.91	-0.24	-0.56	-0.86	-0.85	-1.54
	GPX1	-0.13	-1.34	-1.39	-1.62	-1.45	-0.43	-0.93	-1.08	-1.15	-1.21
	GPX2	-0.19	-0.71	-0.96	-1.48	-1.35	-0.05	-0.99	-1.31	-1.14	-1.48
	HMOX1	0.23	3.00	3.58	3.85	4.18	0.30	2.60	4.05	3.37	3.85
	HSPA1A	0.06	-0.06	-0.02	-0.65	-0.11	0.01	0.09	1.10	1.56	3.13
	NFKB1	-0.04	0.04	0.12	-0.21	0.09	-0.09	-0.06	-0.59	-0.67	-1.30
	NFKB2	0.18	0.88	1.12	1.12	1.36	0.04	0.95	1.07	0.62	0.46
	SOD2	0.02	0.21	0.37	0.53	0.68	-0.19	0.98	1.72	1.19	1.23
Cell cycle regula-tion and apoptosis	*CDKN1A*	-0.07	0.01	0.04	0.00	0.27	-0.10	0.37	0.80	0.67	0.40
	CDKN1B	-0.16	-0.11	-0.23	-0.44	-0.34	-0.25	-0.43	-0.74	-0.86	-1.70
	EF2F1	-0.10	-0.11	-0.20	-0.62	-0.53	-0.12	-0.54	-1.22	-1.31	-2.23
	EGFR	0.03	0.27	0.21	0.11	0.23	-0.24	0.00	-0.54	-0.47	-1.04
	JUN	0.01	0.25	0.18	0.11	0.13	-0.06	0.12	-0.21	-0.45	-1.43
	MYC	0.07	0.14	0.04	-0.31	-0.16	-0.01	0.01	-0.40	-0.76	-1.42
DNA repair and damage response	*BRCA1*	0.01	-0.08	-0.21	-0.49	-0.43	0.03	-0.25	-0.55	-0.62	-1.43
	BRCA2	0.03	0.04	-0.15	-0.44	-0.38	-0.02	-0.14	-0.65	-0.90	-1.70
	DDB2	-0.06	-0.46	-0.34	-0.86	-0.41	-0.12	-0.52	-1.01	-0.99	-1.62
	DDIT3	0.40	0.78	1.34	1.34	2.11	0.10	-0.12	-0.06	-0.56	-0.50
	ERCC4	0.02	0.07	-0.10	-0.21	-0.19	-0.10	-0.16	-0.40	-0.66	-1.28
	GADD45A	0.11	0.20	0.29	0.28	0.51	-0.01	0.25	0.14	-0.25	-1.27
	LIG1	-0.05	-0.17	-0.38	-0.52	-0.55	0.04	-0.14	-0.54	-0.68	-1.42
	LIG3	0.03	-0.08	-0.35	-0.66	-0.52	-0.09	-0.30	-0.64	-0.88	-1.71
	MLH1	-0.04	-0.02	-0.12	-0.36	-0.26	-0.13	-0.13	-0.53	-0.61	-1.07
	MSH2	0.02	-0.14	-0.27	-0.49	-0.49	-0.13	-0.28	-0.34	-0.40	-0.65
	OGG1	0.01	-0.04	-0.14	-0.52	-0.30	-0.05	-0.21	-0.43	-0.48	-0.84
	PARP1	0.04	0.03	-0.03	-0.15	-0.18	-0.06	-0.01	-0.12	-0.37	-0.79
	RAD51	-0.01	0.00	-0.11	-0.25	-0.33	0.02	-0.03	-0.47	-0.50	-0.97
	XPA	0.02	-0.05	-0.16	-0.40	-0.15	-0.20	-0.45	-0.64	-0.79	-1.11
	XPC	-0.07	-0.09	-0.28	-0.30	-0.26	-0.25	-0.13	-0.17	-0.25	-0.83

	XRCC5	-0.01	0.04	-0.13	-0.39	-0.28	-0.06	-0.12	-0.57	-0.45	-0.79
Epigenetic regulation	*DNMT1*	-0.04	-0.09	-0.26	-0.48	-0.54	-0.05	-0.33	-0.50	-0.78	-1.31
	DNMT3a	-0.18	-0.24	-0.37	-0.40	-0.40	-0.11	-0.26	-0.36	-0.55	-0.89
	DNMT3b	-0.01	-0.53	-0.28	-1.01	-0.40	-0.29	-0.59	0.30	-0.10	0.17
	EHMT2	-0.07	0.02	-0.15	-0.49	-0.32	-0.09	-0.20	-0.29	-0.34	-0.69
	EP300	-0.14	-0.23	-0.20	-0.44	-0.12	-0.17	-0.26	-0.36	-0.45	-0.80
	HDAC1	-0.03	-0.23	-0.25	-0.34	-0.32	-0.20	-0.35	-0.38	-0.44	-0.67
	HDAC2	0.06	-0.09	-0.19	-0.60	-0.37	-0.08	-0.36	-0.57	-0.70	-1.12
	HDAC3	-0.05	0.01	-0.07	-0.13	-0.14	-0.20	-0.12	-0.37	-0.48	-0.85
	MBD4	0.00	-0.17	-0.29	-0.36	-0.37	-0.07	-0.21	-0.07	-0.19	-0.56
	MECP2	-0.07	-0.22	-0.29	-0.59	-0.18	-0.15	-0.37	-0.50	-0.66	-1.14
	SETD2	0.02	-0.01	-0.12	-0.38	-0.24	-0.21	-0.26	-0.37	-0.56	-1.15
	TET2	0.02	-0.02	-0.11	-0.39	-0.06	-0.13	-0.35	-0.52	-0.58	-0.97
	TET3	-0.10	0.18	0.03	-0.17	-0.11	-0.33	-0.14	-0.33	-0.74	-1.50

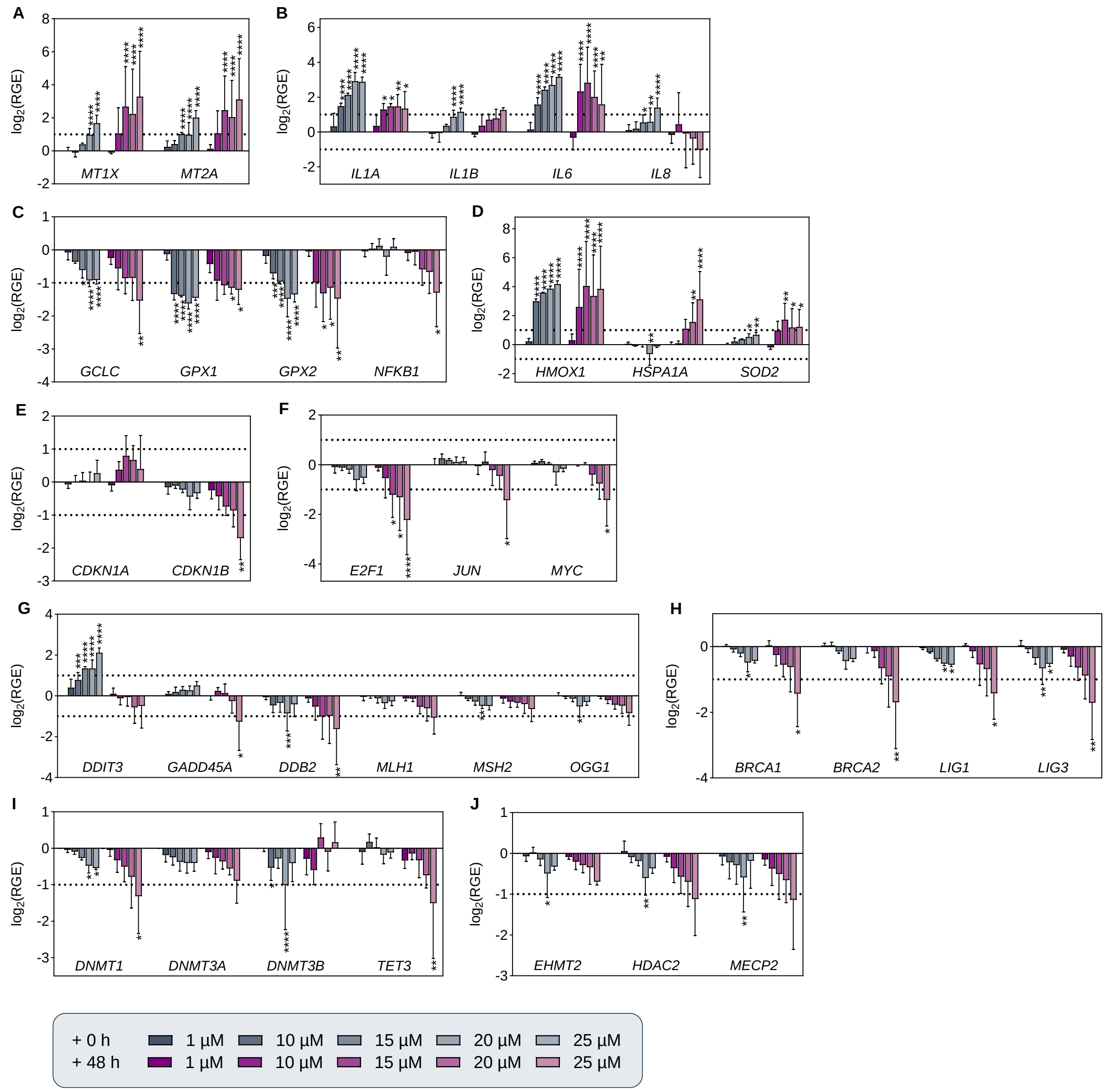

Supplementary Figure S1. Selected gene expression profiles. A549 cells were exposed to $NaAsO_2$ in the dose range of 1-25 µM for 24 h and subsequently subjected to either a 0 h or 48 h arsenite-free recovery phase. Gene expression was determined using high-throughput RT-qPCR. The results are presented as log_2-fold change of the relative gene expression (RGE). Genes involved in metal homeostasis (A), inflammation (B), oxidative stress response (C, D), cell cycle regulation and apoptosis (E, F), DNA repair (G, H), and epigenetic regulation (I, J) were included. Shown are mean values + SD from at least three independent experiments, each performed in duplicate. Statistical analysis was performed using two-way ANOVA followed by Dunnett's post hoc test with respect to all genes to determine the significance of the exposed cells relative to the negative control *($p < 0.05$), ** ($p < 0.01$), *** ($p < 0.001$), **** ($p < 0.0001$).

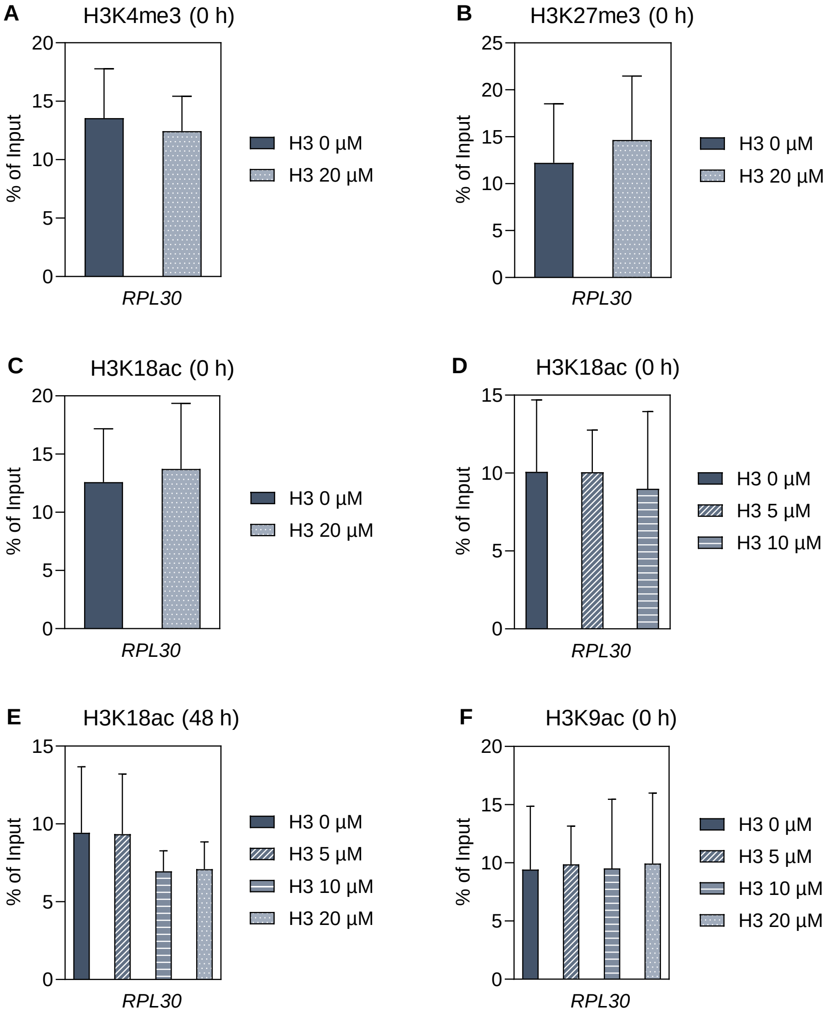

Supplementary Figure S2. ChIP controls of H3 enrichment at the positive control locus *RPL30* across treatment conditions. Shown are the mean values + standard deviation of minimum three independent experiments.

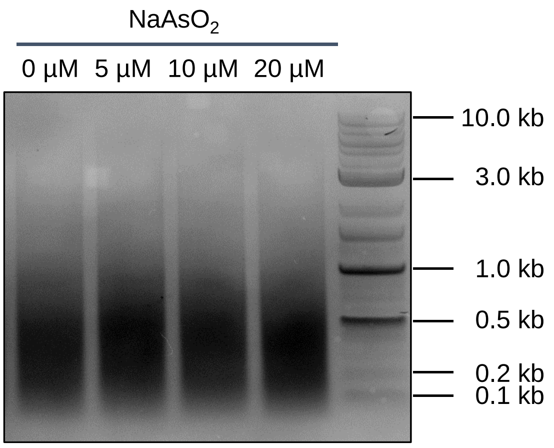

Supplementary Figure S3. Example result of the determination of chromatin fragmentation efficiency in the A549 cells. The DNA fragments were separated gelelectrophoretically on a 2% agarose gel.

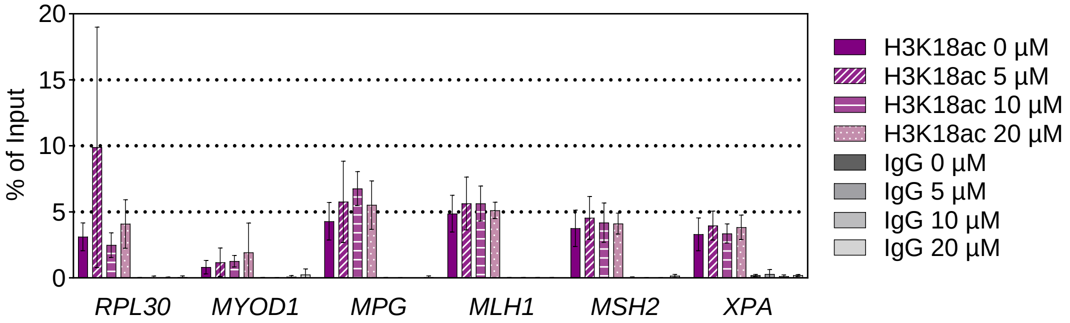

Supplementary Figure S4. Post-incubated H3K18ac levels at selected DNA repair genes and control loci. A549 cells were incubated with 5 µM, 10 µM, and 20 µM $NaAsO_2$ for 24 h and subsequently 48 h post-incubated in the absence of arsenite. *RPL30* was used as a positive control locus, and *MYOD1* as a negative control locus. In addition, an IgG isotype control antibody was used to detect the unspecific background. Shown are the mean values ± standard deviation of at least three independent experiments. Statistical analysis was performed using Welch's test to determine the significance of the treated cells compared to the negative control.

Supplementary Table S3. Detailed information on established and acquired ChIP-qPCR primers.

Gene	RefSeq	Sequence 5'→3'	Product size (bp)	Annealing T_m (°C)
GAPDH	NG_007073.2	fwd: ATGCTGCATTCGCCCTCTTAATGG<br>rev: AGGCGCCCAATACGACCAAATCTA	120	65,2
MB	NG_007075	fwd: CAACCTCTCGGAGCCTCAAT<br>rev: GACTCAGTATATGCCAGGAACCA	136	67,8
MLH1	NG_007109	fwd: CCTTCTCCTTTTCCGCAGAC<br>rev: CCTCCACTTACACTCCAAACAAC	83	67,5
MPG	NC_000016	fwd: TGGCAGTCTCCGTTCTACATT<br>rev: GCAGCACCTAAGTCCTCCT	83	67,8
MSH2	NG_007110	fwd: AAAGGGAGGCAGTCGGAGA<br>rev: GCATGGGAGTAACATCAGAAGGAA	108	68,7
MYOD1		RPL30 SimpleChIP® Human RPL30 Exon 3 Pri-<br>mers #7014 (Cell Signaling Technology)		
RPL30		MYOD1 SimpleChIP® Human MyoD1 Exon 1 Pri-<br>mers #4490 (Cell Signaling Technology)		
XPA	NG_011642	fwd: AGCCTATACTCTGAATCACCAAGC<br>rev: CCGCCTAACTACCTGCTCTC	113	68,3
XPC	NG_011763	fwd: GTTGTGCTCTTTCCTGCTTCC<br>rev: GCCTAGTCACGCCCCTAAA	80	68,2
XRCC1	NG_033799	fwd: TGGAAGTCTAAGGAGAGTCATGG<br>rev: CCGTGGAAGTTCACCTATGG	158	66,7

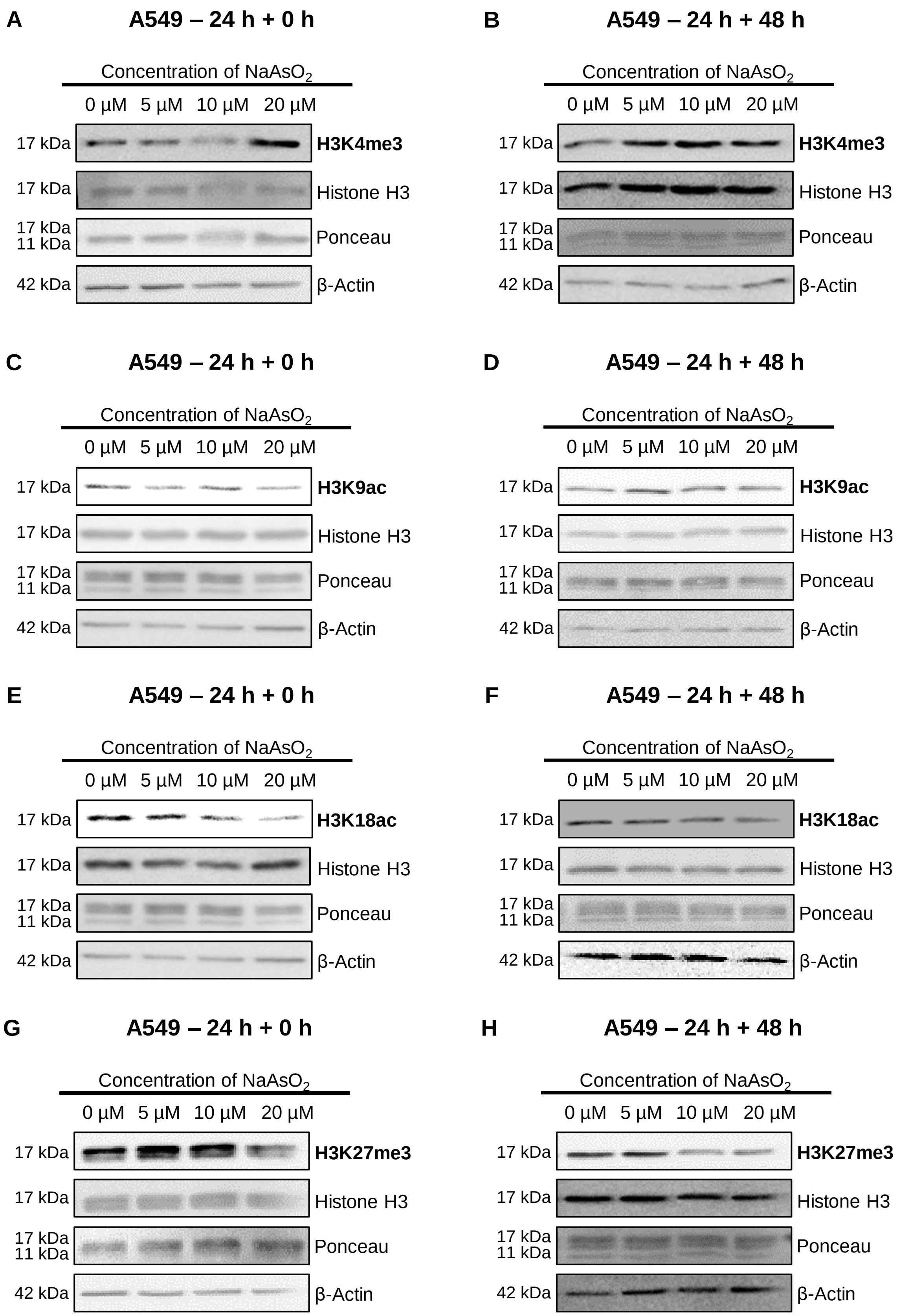

Supplementary Figure S5. Example blots of the respective global hPTM determinations in A549 cells. Cells were exposed to $NaAsO_2$ for 24 h and subsequently subjected to either a 0 h (A, C, E, G) or 48 h (B, D, F, H) arsenite-free recovery period.

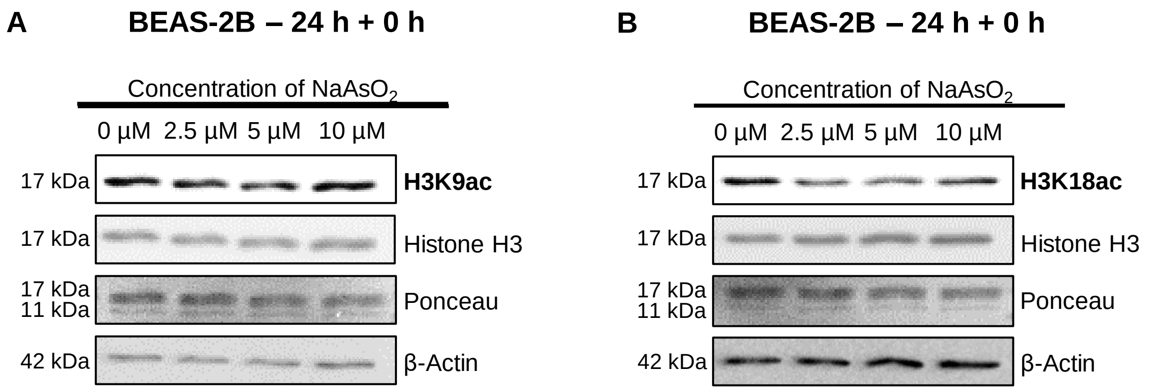

Supplementary Figure S6. Example blots of the respective global hPTM determinations in BEAS-2B cells. Cells were exposed to $NaAsO_2$ for 24 h.

Supplementary Table S4. Liquid Chromatography (LC) gradient for histone separation on the C18 column with a flow rate of 300 nL/min.

Time (min)	Gradient (% solvent B)
0	2.5
1.5	2.5
7	8.8
62	38.8
70	51.3
71	90.3
80	90.3

Supplementary Table S5. Acquisition parameters used on the Orbitrap Ascend Tribid MS instrument for Data-Dependent Acquisition (DDA).

Global	Polarity	positive
	Fragmentation	HCD
	Range	300 to 1300 m/z
Full MS Scans	Resolution	120 000
	Absolute AGC	4e5
	Max inj time	Auto
	Dynamic Exclusion	10 s
	Isolation mode	Quadrupole
MS/MS Scans	Resolution	30 000
	Absolute AGC	5e4
	Max inj time	59 ms
	NCE	30
	Isolation window	1.5 m/z

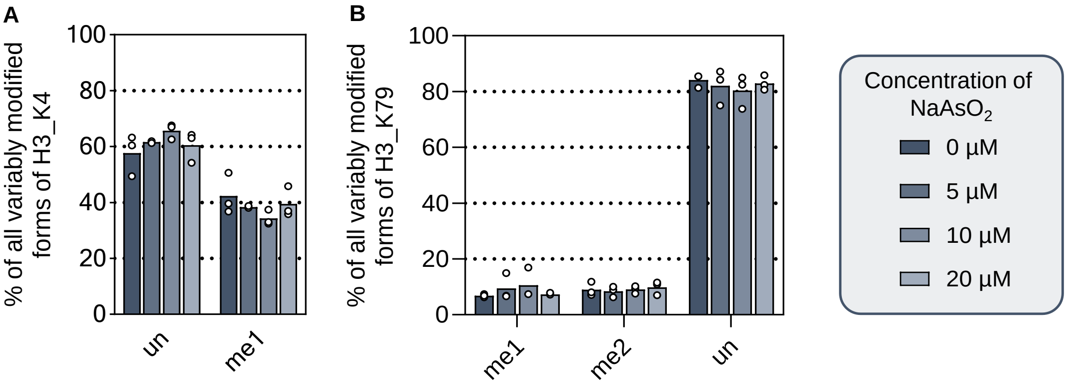

Supplementary Figure S7. Additional results of the histone landscape profiling. A549 cells were incubated with increasing $NaAsO_2$ concentrations. hPTMs were analyzed by LC-MS/MS. Shown are the modified forms of H3_K4 (A) and H3_K79 (B). The quantitative data obtained from biological triplicates were plotted as individual dots. The bar heights correspond to the mean values.

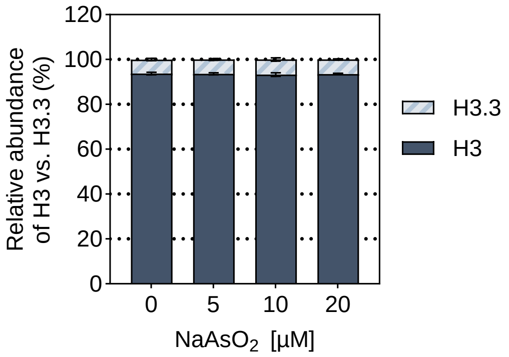

Supplementary Figure S8. Effect of arsenite on the relative abundance of histone H3 vs. H3.3. A549 cells were treated with $NaAsO_2$ for 24 h. Histones were isolated and analyzed by LC-MS/MS. Shown are the means ± 95% confidence intervals of all summed MS signals of variably modified K27-R40 peptide sequences from H3 compared to H3.3. Data were obtained from biological triplicates.
